# HSPA1A inhibits pyroptosis and neuroinflammation after spinal cord injury via DUSP1 inhibition of the MAPK signaling pathway

**DOI:** 10.1186/s10020-025-01086-9

**Published:** 2025-02-09

**Authors:** Xuegang He, Bo Deng, Cangyu Zhang, Guangzhi Zhang, Fengguang Yang, Daxue Zhu, Yong Yang, Bing Ma, Xuchang Hu, Yonggang Wang, Xuewen Kang

**Affiliations:** 1 Department of Orthopedics, Lanzhou University Second Hospital, Lanzhou, 730000 Gansu China; 2 Department of Spine Surgery, Honghui Hospital, Xi’an Jiaotong University, Xi’an, 710054 China; 3 Orthopedics Key Laboratory of Gansu Province, Lanzhou, 730000 Gansu China

**Keywords:** Traumatic spinal cord injury, HSPA1A, DUSP1, Pyroptosis, Inflammation

## Abstract

**Background:**

Inflammation and proinflammatory programmed cell death, referred to as pyroptosis, are important causes of poor functional recovery after traumatic spinal cord injury (TSCI). Heat shock protein family A member 1A (HSPA1A) is a molecular chaperone protein that is highly expressed after TSCI and is thought to be neuroprotective. However, the mechanisms underlying the protective effects of HSPA1A after TSCI are unclear.

**Methods:**

The levels of pyroptosis and inflammation after TSCI were determined by enzyme-linked immunosorbent assay (ELISA) and western blotting analysis. The role of HSPA1A in regulating pyroptosis and inflammation in TSCI was verified by in vivo and in vitro experiments. The molecular mechanism of the effects of HSPA1A in TSCI was elucidated by bioinformatics and coimmunoprecipitation analyses.

**Results:**

Pyroptosis and inflammation are significantly increased after TSCI. HSPA1A overexpression in microglia attenuated nigericin- and lipopolysaccharide (LPS)-induced pyroptosis and inflammation in vitro, whereas knockdown of HSPA1A aggravated pyroptosis and inflammation. In vivo, overexpression of HSPA1A reduced tissue damage, nerve cell death, pyroptosis, and inflammation in TSCI rats and promoted functional recovery. Mechanistically, we identified that HSPA1A interacts with dual specificity phosphatase 1 (DUSP1) and inhibits activation of the mitogen-activated protein kinase (MAPK) pathway, thereby attenuating pyroptosis and inflammation.

**Conclusion:**

HSPA1A reduces pyroptosis and inflammation after TSCI by upregulating DUSP1 and inhibiting MAPK pathway activation. HSPA1A activation has potential as a therapeutic approach to promote functional recovery after TSCI.

**Supplementary Information:**

The online version contains supplementary material available at 10.1186/s10020-025-01086-9.

## Introduction

Traumatic spinal cord injury (TSCI) is a common central nervous system (CNS) disease that is usually caused by direct or indirect damage to spinal cord tissue from broken bone fragments, intervertebral discs, or ligaments resulting from traumatic external forces (e.g., WOUNDS, GUNSHOT; WHIPLASH INJURIES; etc.), resulting in impaired or even loss of sensory and motor as well as autonomic function below the plane of injury (Maynard et al. 2023). Due to the difficulty of treatment, TSCI leads to devastating consequences of physical disability, psychological disorders, and social burden (You et al. 2023). The pathogenesis of TSCI includes primary mechanical injury and secondary injury (Alizadeh et al. 2019). Mechanical injury is irreversible, and in addition to early surgical decompression, the main direction of research for the treatment of TSCI involves limiting the development of secondary injuries initiated by the primary injury, such as interventions against secondary spinal cell death, inflammation, oxidative stress, demyelination and axon degeneration (Peng et al. 2023; Lu et al. 2023; Yuan et al. 2023; Xuan et al. 2023; Li et al. 2023).

TSCI produces cell debris and the release of intracellular contents, which act as powerful inflammatory stimuli to trigger inflammation and further induce nerve cell death (He et al. 2022). Pyroptosis is a newly identified form of programmed cell death that is closely associated with inflammation and is mainly regulated by gasdermin D (GSDMD) and cysteinyl aspartate-specific proteinases (caspases) (Jiang et al. 2023; Shi et al. 2015 Oct 29). Inflammasomes (e.g., NOD-like receptor thermal protein domain-associated protein 3, NLRP3) are activated and released after TSCI (Mortezaee et al. 2018), and recruit and activate pro-caspase-1 via apoptosis-associated speck-like protein containing a caspase recruitment domain (ASC). Activated caspase-1 substrates are also involved in the cleavage of GSDMD to produce the N-terminal fragment of GSDMD (GSDMD-NT), which inserts pores in the cell membrane and compromises membrane integrity, accompanied by the secretion of inflammatory signaling molecules, including interleukin (IL)-1β/18 (Müller et al. 2023). In addition, lipopolysaccharide (LPS) can activate human caspase-4/5 or mouse caspase-11, and similar to caspase-1, activated caspase-4/5/11 is able to cleave GSDMD, further inducing pyroptosis (Shi et al. 2015). The present study showed that spinal cord cellular pyroptosis is significantly increased after TSCI and is an important cause of aggravated neuroinflammation and cell death (Dai et al. 2019; Al Mamun et al. 2021). Therefore, inhibition of pyroptosis and excessive inflammation represents a potential strategy for the treatment of TSCI.

Heat shock protein family A member 1A (HSPA1A) is a member of the HSP1 protein family encoded by the HSPA1A gene, also known as inducible HSP70 or HSP72, and is a major cytoprotective HSP70 protein (Wu et al. 2021). The expression of HSPA1A is increased in many diseases and pathological processes, and induction of HSPA1A can effectively slow the progression of several diseases (Hu et al. 2022). HSPA1A has been shown to interfere with multiple cell death pathways, to inhibit inflammation, and to have a promising neuroprotective effect in central nervous system (CNS) trauma (Demyanenko et al. 2021; Li et al. 2019). Our previous study demonstrated that HSPA1A expression was elevated in the acute TSCI stage and that further promotion of its expression inhibited secondary neuronal apoptosis (He et al. 2023a). However, it is not clear whether HSPA1A can inhibit pyroptosis and inflammation after TSCI.

The mitogen-activated protein kinase (MAPK) pathway is involved in the regulation of pyroptosis and inflammation in CNS injury (Chen et al. 2023a; Xu et al. 2021a; Liu et al. 2021a); the pathway is shared by three distinct cascades named according to their MAPK layer components: extracellular signal-regulated kinase (ERK), p38 MAPK, and c-Jun N-terminal kinase (JNK) (Phan et al. 2023). The MAPK pathway can be activated in response to cellular stress, modulating the production of multiple inflammatory factors and promoting pyroptosis (Kudaravalli et al. 2022; Song et al. 2022). Therefore, inhibition of its activation has potential to suppress pyroptosis and inflammation. Dual specificity phosphatase (DUSP) can negatively regulate the activation of MAPKs through dephosphorylation of phosphotyrosine and phosphothreonine residues (Keyse 2000). Numerous studies have shown that DUSP1 can inhibit MAPK phosphorylation to suppress the production of inflammatory factors (Hoppstädter and Ammit 2019). However, whether DUSP1can inhibit pyroptosis and suppress inflammation after TSCI has not been investigated.

HSPA1A has been shown to inhibit cell death and inflammation, but whether HSPA1A can inhibit pyroptosis and inflammation after TSCI and the detailed underlying molecular mechanisms have not been reported. In the present study, we showed that HSPA1A regulates pyroptosis and inflammation and has a significant neuroprotective effect in SCI rats. Microglia are important cells in the innate immune response after CNS trauma and are critical for the subsequent inflammatory response. There is evidence that pyroptosis occurs predominantly in microglia in CNS neuroimmune diseases (Xu et al. 2021b). Therefore, in the present study, we established an in vitro microglial pyroptosis and inflammation model. Through in vivo and in vitro experiments, we demonstrated that HSPA1A inhibited pyroptosis and inflammation after TSCI by regulating the expression of DUSP1 and further inhibiting activation of the MAPK pathway. This study elucidated the molecular mechanism underlying the neuroprotective effect of HSPA1A in SCI rats and identified a potential therapeutic target for the treatment of TSCI.

## Materials and methods

### Animals and SCI model construction

All experimental protocols were approved by the Animal Ethics Committee of Lanzhou University Second Hospital and applied in accordance with relevant guidelines (approval number: D2022-162). Adult female Sprague–Dawley rats (200–250 g) were provided by the Experimental Animal Center of Lanzhou University. Prior to the experiment, three rats per cage were kept for 1 week in a specific pathogen-free environment maintained at 22 °C–26 °C under a 12-h light/12-h dark photoperiod, with free access to food and water. The surgical procedure for SCI was performed as described in our previous report (He et al. 2023a). The rats were anesthetized by intraperitoneal injection of 1% pentobarbital sodium while in the prone position. The skin above the 8th thoracic vertebra of each rat was cleaned, incised, and exposed layer by layer. The lamina was then detached, and the T10 spinal cord segment was exposed. Next, the T10 spinal cord center was struck with a 10-g weight from a height of 25 mm, in a modification of Allen’s method. Immediately after the blow, the hind limbs of each rat were observed to spasm and twitch, indicating that the contusion model was successful. Then, the wound was sutured. The entire operative process was performed in a sterile environment, and no painkillers or anti-inflammatory drugs were used after the operation to exclude the influence of the experimental results. Bladder massage was performed twice per day to assist urination until the rats could void spontaneously. The rats were divided into a sham group (simple laminal exposure, no spinal cord contusion, *n* = 10 rats), SCI group (exposed spinal cord, contusion, *n* = 10 rats), and HSPA1A overexpression group (HSPA1A-overexpressing lentivirus was injected intrathecally once a day for 3 days following SCI, *n* = 10 rats). The spinal cord tissue was removed 7 days after injury for follow-up experiments.

### Assessment of locomotor capacity

The Basso–Beattie–Bresnahan (BBB) scoring method was used to evaluate motor function. This method comprehensively assesses rat motor function by measuring hind limb joint activities and range of motion, load-bearing capacity, coordination of the front and rear limbs, and movement of the front and rear paws and tail (Basso et al. 1995). All rats were acclimatized in an open field environment for 1 h before functional behavioral assessment. The rats were then observed for body, tail, and hind limb movements and scored according to the criteria in the BBB scoring scale. Scoring was performed before surgery and daily after injury until sacrifice.

### Footprint analysis

On day 7 after surgery, the forefeet and hindfeet of the rats were coated with blue and red dye, respectively, and the rats were made to walk in an extended straight line on a clean sheet of paper to record the footprints. The distance between two footsteps on the same side during continuous walking was measured and analyzed in each group.

### Preparation of tissues and hematoxylin and eosin and Nissl staining

On day 7 after surgery, the rats were anesthetized with 1% pentobarbital sodium, and then after systemic irrigation using phosphate-buffered saline (PBS) or 4% paraformaldehyde, spinal cord tissues were isolated, fixed in 4% paraformaldehyde for 24 h, and embedded in paraffin; transverse Sects. 4 μm thick were prepared and then baked, dewaxed, and rehydrated by passage through a graded ethanol series. Hematoxylin and eosin (HE) and Nissl staining were performed using commercial kits according to the manufacturer’s instructions (Solarbio, Beijing, China, cat. G1120). Spinal cord morphology and nissl bodies were examined under a light microscope (Olympus, Tokyo, Japan).

### Enzyme-linked immunosorbent assay

Samples of 1 mg of spinal cord tissue were weighed, placed in 2 ml of precooled PBS, and ground well with a tissue homogenizer; all operations were performed on ice. The grinding solution was centrifuged at 4 °C and 13,000 rpm for 20 min, and the supernatant was collected for subsequent assays. The levels of IL-1β, IL-18, IL-6, IL-10, and tumor necrosis factor (TNF)-α were determined separately in each group using commercials kits in accordance with the manufacturer’s instructions (NeoBioscience, Shenzhen, China, cat. ERC007.96, ERC010.96, ERC003.96, ERC004.96 and ERC102a.96). For in vitro experiments, microglial culture medium was collected and centrifuged at 4 °C and 1500 rpm for 10 min, and the supernatant was collected for subsequent assays. The levels of IL-1β, IL-18, IL-6, IL-10, and TNF-α were determined separately in each group using commercials kits in accordance with the manufacturer’s instructions.

### Immunohistochemical staining

Spinal cord tissue sections were baked, deparaffinized, and rehydrated by passage through a graded ethanol series, followed by antigen retrieval with sodium citrate. Next, the sections were blocked with goat serum for 1 h and incubated with specific antibodies overnight at 4 °C. The sections were then developed using 3,3′-diaminobenzidine (DAB) solution (Gene-Tech, Shanghai, China, cat. GK800511) before counterstaining with hematoxylin (Solarbio) and examined under a light microscope (Olympus).

### Immunofluorescence staining

For tissue immunofluorescence staining, tissue sections were baked, deparaffinized, and rehydrated by passage through a graded ethanol series, followed by antigen retrieval with sodium citrate. Next, the sections were blocked with goat serum (Solarbio) for 1 h and incubated with specific antibodies overnight at 4 °C. The sections were subsequently incubated with Alexa 488- or Alexa 594-labeled secondary antibody (Proteintech, Wuhan, China, cat. SA00013-2 and SA00013-3) for 1 h at room temperature, and the nuclei were stained with 4′,6-diamidino-2-phenylindole (DAPI; Solarbio, cat. S2110) and observed using a fluorescence microscope (Olympus). For immunofluorescence staining, cells were seeded onto coverslips, fixed in 4% paraformaldehyde for 30 min at room temperature, permeabilized in 0.5% Triton X-100 (Beyotime, Shanghai, China, cat. P0096) for 15 min, and blocked with goat serum (Solarbio, cat. SL038) for 1 h. The subsequent steps were as described for tissue immunofluorescence.

### Gene set enrichment analysis, Kyoto encyclopedia of genes and genomes (KEGG) analysis, and protein–protein interaction analysis

We downloaded the rat SCI gene expression profile dataset GSE464 (https://www.ncbi.nlm.nih.gov/geo/query/acc.cgi?acc = GSE464) from the Gene Expression Omnibus (GEO) database (https://www.ncbi.nlm.nih.gov/) as mentioned in our previous report (He et al. 2023a). Gene set enrichment analysis (GSEA, http://software.broadinstitute.org/gsea/index.jsp) was performed to examine the biological functions of differentially expressed genes (DEGs) between SCI group and Sham group. A false detection rate (FDR) q value < 25%, *P* < 0.05, and normalized enrichment score (NSE) > 1 or < − 1 were selected to sort the pathways enriched in each phenotype (Zha et al. 2023). We used the *limma* R package to analyze the DEGs between the SCI and sham groups. The DEGs were then imported into the DAVID database for Kyoto Encyclopedia of Genes and Genomes (KEGG) pathway enrichment analysis. The cutoff value for significant gene enrichment was *P* < 0.05. Enrichment results containing HSPA1A were visualized using the Bioinformatics website (http://www.bioinformatics.com.cn/). In addition, the DEGs were imported into the STRING database for protein–protein interaction (PPI) analysis; the minimum interaction score was set to 0.7, and the PPI network results were visualized with Cytoscape v3.9.1 (https://www.cytoscape.org/) (He et al. 2023b).

### Coimmunoprecipitation

Coimmunoprecipitation (co-IP) was performed with a Beaver Beads™ Protein A/G Matrix Immunoprecipitation kit (Beaver, Suzhou, China, cat. 22,202–20) in accordance with the manufacturer’s instructions. The spinal cord tissue was lysed with radio immunoprecipitation assay (RIPA) lysis buffer (Beyotime, cat. P0013B) supplemented with phenylmethylsulfonyl fluoride (PMSF) (Beyotime, cat. ST2573). The lysate was sonicated for 10 s, kept on ice for 20 min, centrifuged, and the supernatant was collected. Protein A/G magnetic beads (50 µl) were mixed into the protein–antibody solution and incubated on a shaker at 4 °C for 12 h. The magnetic beads were precipitated with a magnetic stand. The magnetic beads were boiled with SDS loading buffer and subjected to immunoblotting analysis.

### Primary cell isolation and culture

Whole brains of neonatal Sprague–Dawley rats (days 0–2) were dissected out, and the meningeal layer and blood vessels were removed. The cortex was then digested with trypsin for 15 min at 37 °C, and the digestion was terminated by adding an equal volume of complete medium consisting of high-glucose Dulbecco’s modified Eagle’s medium (DMEM; Gibco-BRL, Grand Island, NY, USA, cat. 11,965,092) supplemented with 10% fetal bovine serum (FBS; FuHeng, Shanghai, China, cat. FH100-900) and 1% penicillin/streptomycin (Gibco-BRL, cat. 15,070,063). Mixed neuroglia was obtained and cultured in complete medium at 37 °C in a humidified 5% CO_2_ atmosphere. The complete medium was changed every 3 days. Microglia grew in the upper layer of the mixed neuroglia and were easily detached; therefore, the mixed cells were gently shaken between days 7 and 14 of culture to isolate and purify primary microglia. Primary microglia were collected and cultured using complete medium, which was changed every 3 days.

### Cell culture and treatment

The highly aggressively proliferating immortalized (HAPI) microglia used in this study were provided by YaJi Biological Co., Ltd. (Shanghai, China cat. YS1284C) and cultured in complete medium at 37 °C in a humidified 5% CO_2_ atmosphere. The complete medium was changed every 3 days. LPS (Thermo Fisher Scientific, Waltham, MA, USA, cat. 00–4976-93) and nigericin (an inducer of NLRP3; MCE, Shanghai, China, cat. 28,380–24-7) were used to establish models of pyroptosis and inflammation in primary and HAPI microglia. The specific methods were as follows. LPS was added at 1 μg/ml as pretreatment for 5 h, and then 10 μM nigericin was added for induction (Sheng et al. 2021). HSPA1A-overexpressing lentivirus was purchased from GeneChem (Shanghai, China), and HSPA1A short hairpin RNA (shRNA) was purchased from GenePharma (Suzhou, China). Lentivirus was transfected at a multiplicity of infection of 100 in serum-free DMEM. After 24 h, the supernatant was replaced with complete medium. Inhibition of DUSP1 was performed using inhibitor (E)-2-benzylidene-3-(cyclohexylamino)-2,3-dihydro-1H-inden-1-one (BCI) (1 nM for 24 h).

### Transmission electron microscopy

Cells were collected by centrifugation (1500 rpm for 10 min at 4 °C) and fixed in 2.5% glutaraldehyde for 24 h, followed by fixation with 1% osmium tetroxide for 2 h at 4 °C. The cells were then dehydrated by passage through an ascending ethanol series and permeated with Spurr’s resin. Then, the cells were cut into ultrathin sections at a thickness of approximately 70 nm and stained with 2% uranyl acetate for 10 min. The sections were then stained with 0.3% lead citrate for 10 min and rinsed three times with distilled water. After thorough drying in air, the sections were observed under a transmission electron microscope (Hitachi, Tokyo, Japan).

### Western blotting analysis

Protein was extracted from spinal cord tissue and microglia for western blotting analysis using RIPA lysis buffer (Beyotime). The supernatant was subjected to protein assay using a Bradford protein assay kit (Thermo Fisher Scientific, cat. 23,236). Protein samples were separated by 10% sodium dodecyl sulfate‒polyacrylamide gel electrophoresis (SDS-PAGE) and transferred onto polyvinylidene fluoride membranes (Millipore, Billerica, MA, USA, cat. IPFL00010). The membranes were blocked with 5% bovine serum albumin for 2 h at room temperature, and incubated overnight at 4 °C with primary antibodies against the following proteins: HSPA1A (anti-rabbit, 1:1000; Proteintech, cat. 25,405–1-AP), DUSP1 (anti-rabbit, 1:1000; ImmunoWay, Plano, TX, USA, cat. YT2771), Neuronal nuclei antigen (NeuN, anti-rabbit, 1:1000; Proteintech, cat. 26,975–1-AP), Glial fibrillary acidic protein (GFAP, anti-rabbit, 1:1000; Proteintech, cat. 16,825–1-AP), Neurofilament Protein (NF-200, anti-rabbit, 1:1000; Proteintech, cat. 18,934–1-AP), Ionized calcium binding adaptor molecule 1 (Iba-1, anti-rabbit, 1:1000; Proteintech, cat. 10,904–1-AP), NLRP3 (anti-rabbit, 1:1000; Proteintech, cat. 27,458–1-AP), ASC (anti-rabbit, 1:1000; ABclonal, Wuhan, China, cat. A22046), Caspase-1 (anti-rat,1:1000; ABclonal, cat. A21085), GSDMD-NT (anti-rabbit, 1:1000; ABclonal, cat. A10164), Inducible nitric oxide synthase (iNOS, anti-rabbit, 1:1000; Proteintech, cat. 22,226–1-AP), Cyclooxygenase 2 (COX-2, anti-rabbit, 1:1000; ABclonal, cat. A1253), TNF-α (anti-rabbit, 1:1000; Proteintech, cat. 17,590–1-AP), p38 (anti-rabbit, 1:1000; Proteintech, cat. 14,064–1-AP), p-p38 (anti-rabbit, 1:1000; Proteintech, cat. 28,796–1-AP), JNK (anti-rabbit, 1:1000; Proteintech, cat. 24,164–1-AP), p-JNK (anti-rabbit, 1:1000; Proteintech, cat. 80,024–1-RR), ERK1/2 (anti-rabbit, 1:1000; Proteintech, cat. 11,257–1-AP), p-ERK1/2 (anti-rabbit, 1:1000; Proteintech, cat. 28,733–1-AP), and β-actin (anti- rabbit, 1:2000; Proteintech, cat. 81,115–1-RR). The membranes were then washed three times (5 min each time) and incubated with horseradish peroxidase-conjugated goat anti-rabbit antibody (1:5000; Beyotime, cat. A0277) or horseradish peroxidase-conjugated goat anti-mouse antibody (1:5000; Beyotime, cat. A0216) at room temperature for 1 h. Protein signals were visualized using an enhanced chemiluminescence kit (Bio-Rad, Hercules, CA, USA,cat. 1,705,061) and the ChemiDoc XRS imaging system (Bio-Rad). ImageJ software was used to analyze the western blotting data.

### Statistical analysis

Data were analyzed using GraphPad Prism 9 (GraphPad Software Inc., San Diego, CA, USA). All data are shown as the mean ± standard error of the mean (SEM). Pairs of groups were compared using Student’s *t* test, and more than two groups were compared using one-way analysis of variance (ANOVA). In all analyses, *P* < 0.05 was taken to indicate statistical significance. All experiments were repeated independently at least three times.

## Results

### Pyroptosis and inflammation levels were enhanced after SCI

We established a rat SCI model (Fig. S1A) and examined the expression levels of pyroptosis- and inflammation-related proteins in the spinal cord tissues of the Sham group and SCI group. Western blotting analysis showed that the expression levels of the pyroptosis-related proteins NLRP3, ASC, Caspase-1, and GSDMD-NT were elevated in the SCI group (Fig. S1C). Enzyme-linked immunosorbent assay (ELISA) showed that the levels of IL-1β and IL-18 were significantly elevated in the SCI group (Fig. S1H, I). Meanwhile, the expression levels of the inflammation-related proteins iNOS (is a two-domain enzyme that is induced by multiple cell types, including microglia, in response to the production of inflammatory factors and is an inflammatory marker.), COX-2 (is an important enzyme in the body. It is induced during inflammation and pain and can be used as a marker for detecting inflammation.), and TNF-α were elevated in the SCI group (Fig. S1J). The levels of the inflammatory factors IL-6 and TNF-α and the anti-inflammatory factor IL-10 (is an anti-inflammatory cytokine with multiple, pleiotropic, effects in immunoregulation and inflammation, and can inhibit the secretion of pro-inflammatory cytokines.) were elevated in the SCI group (Fig. S1N–P). These results indicated that pyroptosis and inflammation were significantly enhanced after SCI in rats.

### HSPA1A alleviated pathophysiological damage to spinal cord tissue and promoted functional recovery in SCI rats

**Fig. 1:**
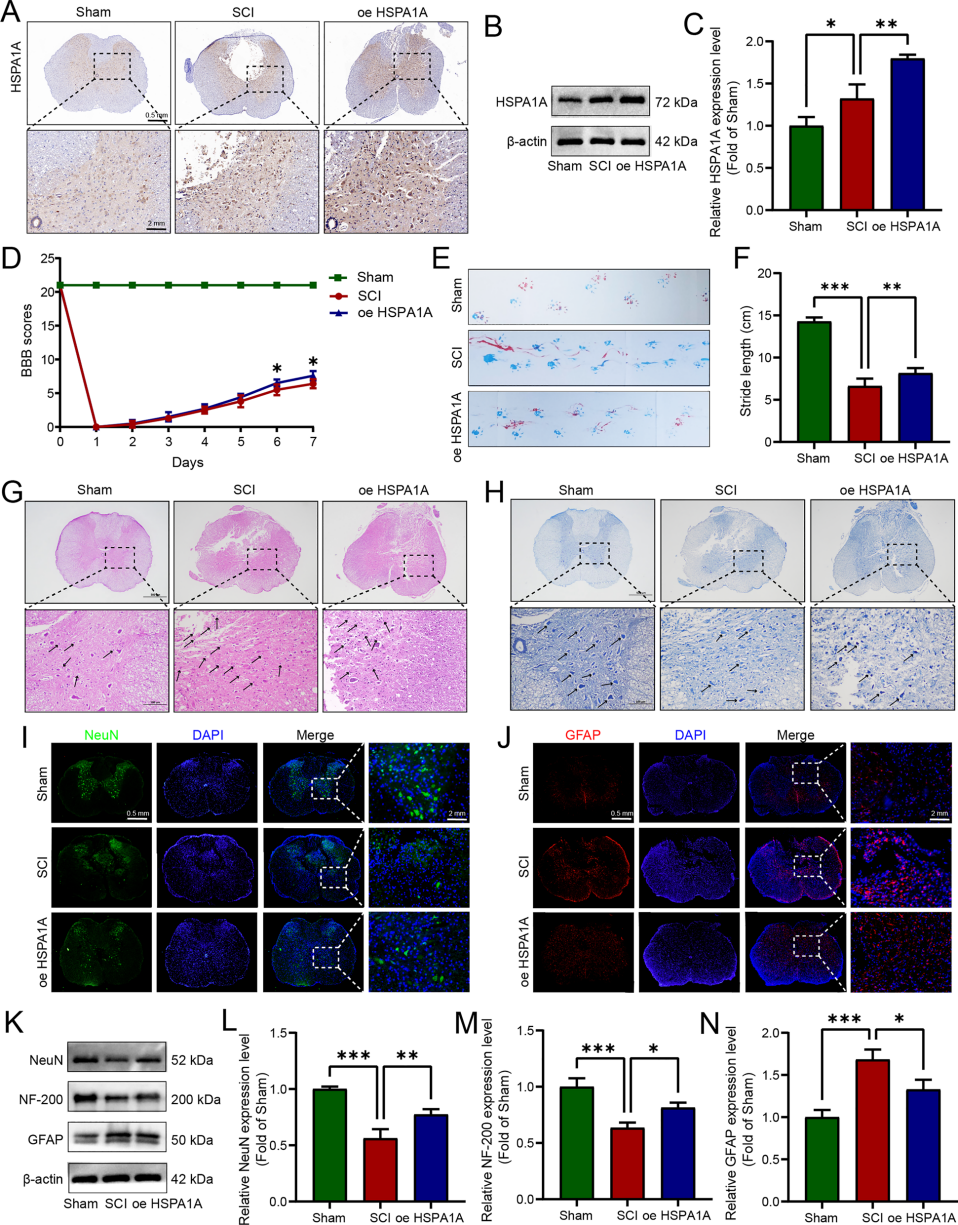
HSPA1A alleviated pathophysiological damage to spinal cord tissue and promoted functional recovery in SCI rats. **A** Representative immunohistochemistry images of HSPA1A in the spinal cord on the 7th day after surgery in each group. **B** Western blotting analysis of HSPA1A expression levels in each group. **C** Quantitative analysis of HSPA1A levels. **D** BBB scores of rats in each group. **E** Representative images of footprint analysis on the 7th day after surgery, rat forefoot in blue, hindfoot in red. **F** Quantification analysis of footprint stride length. **G** Representative HE images of HSPA1A in the spinal cord on the 7th day after surgery in each group. The black arrow points to inflammatory cells. **H** Representative Nissl images of HSPA1A in the spinal cord on the 7th day after surgery in each group. The black arrow points to nissl bodies. **I** Representative immunofluorescence images of NeuN (green) in the spinal cord at 7 d after surgery in each group. **J** Representative immunofluorescence images of GFAP (red) in the spinal cord on the 7th day after surgery in each group. **K** Western blotting analysis of NeuN, NF-200 and GFAP expression levels in each group. **L** Quantitative analysis of NeuN levels. **M** Quantitative analysis of NF-200 levels. **N** Quantitative analysis of GFAP levels. (*P < 0.05, **P < 0.01 and ***P < 0.001)

To simulate TSCI, a modified Allen’s strike rat SCI model and intervention involving treatment with HSPA1A-overexpressing lentivirus were used in this study. As shown in Fig. 1A, after SCI, the expression of HSPA1A was significantly increased at the injury site, and the results of western blotting analysis were consistent with those of immunohistochemistry (Fig. 1B, C). BBB scores and footprint analysis indicated that HSPA1A overexpression promoted the recovery of motor function in SCI rats (Fig. 1D–F). Nissl bodies are a type of basophilic substance in the cytoplasm of neurons, they are widely distributed in neurons and can be stained with basic dyes. Nissl staining can be used to determine the extent of neuronal damage. HE and Nissl staining were used to examine the effects of HSPA1A on the tissue at the injury site, and the results are shown in Fig. 1G, H. In the Sham group, the spinal cord tissue was intact, the nerve fibers were regularly aligned, the nissl bodies were intact in morphology, and no inflammatory cell infiltration was observed. However, in the SCI group, the spinal cord was disorganized, the nerve cells were destroyed, the nissl bodies were fragmented, and inflammatory cell infiltration was observed. Compared with the SCI group, the neuronal damage and histological changes in the HSPA1A overexpression group were significantly improved, and the number of nissl bodies was numerous. We then used immunofluorescence to detect NeuN (a neuronal marker protein) and GFAP (a marker of astrocyte activation, is overexpress during first and second activation of astrocytes). The results are shown in Fig. 1I, J. After SCI, the expression of NeuN in the anterior horn was significantly reduced, while it was significantly increased in the HSPA1A overexpression group. Expression of GFAP was increased around the region of the injury in the SCI group but significantly decreased in the overexpression group. We also examined the expression of NeuN, NF200, and GFAP in the spinal cord tissues of each group by western blotting. The SCI group showed decreased expression of NeuN and NF200 and increased expression of GFAP compared with the Sham group, whereas the HSPA1A overexpression group showed restoration of the levels of NeuN and NF200 compared with the SCI group and a significant decrease in GFAP expression compared with the SCI group (Fig. 1K–N). In summary, these results showed that HSPA1A overexpression alleviated pathophysiological damage to spinal cord tissue and promoted functional recovery in SCI rats.

### HSPA1A overexpression inhibited pyroptosis in SCI rats

**Fig. 2:**
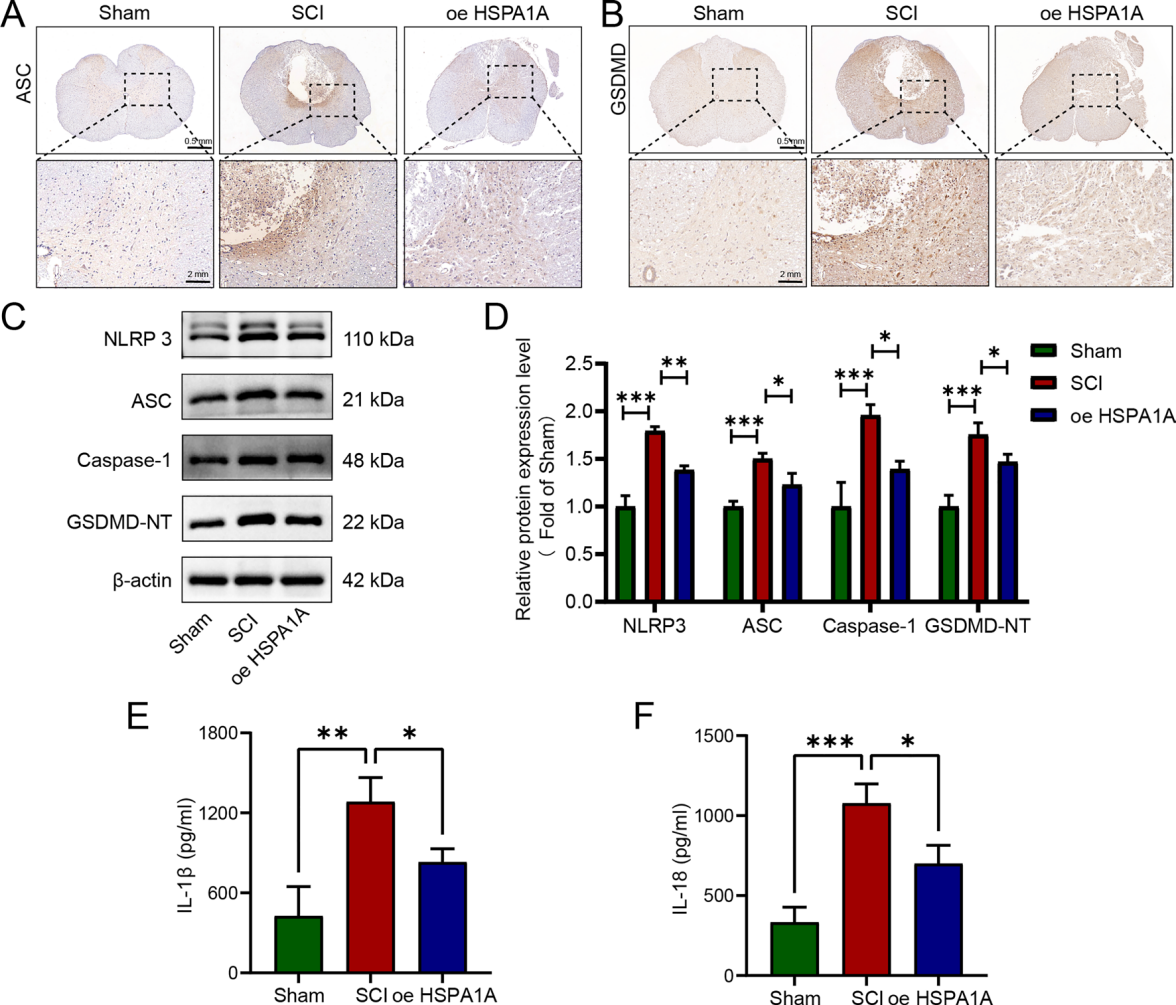
HSPA1A overexpression inhibited pyroptosis in SCI rats. **A** Representative immunohistochemistry images of ASC in the spinal cord on the 7th day after surgery in each group. **B** Representative immunohistochemistry images of GSDMD in the spinal cord on the 7th day after surgery in each group. **C** Western blotting analysis of NLRP3, ASC, Caspase-1 and GSDMD-NT expression levels in each group. **D** Quantitative analysis of NLRP3, ASC, Caspase-1 and GSDMD-NT levels. **E** ELISA analysis of IL-1β levels in each group. **F** ELISA analysis of IL-18 levels in each group. (*P  <0.05, **P <0.01, ***P <0.001)

Next, we explored the effects of HSPA1A overexpression in vivo on pyroptosis in SCI rats. Immunohistochemical analysis showed that after SCI, the expression of ASC and GSDMD increased significantly, and HSPA1A overexpression inhibited expression of these proteins (Fig. 2A, B). Meanwhile, western blotting showed that HSPA1A overexpression reduced the expression of NLRP3, ASC, Caspase-1, and GSDMD-NT after SCI (Fig. 2C, D). The ELISA results showed that HSPA1A overexpression reduced the levels of IL-1β and IL-18 after SCI (Fig. 2E, F). The above results indicated that HSPA1A inhibited pyroptosis in SCI rats.

### HSPA1A overexpression inhibited inflammation in SCI rats

**Fig. 3:**
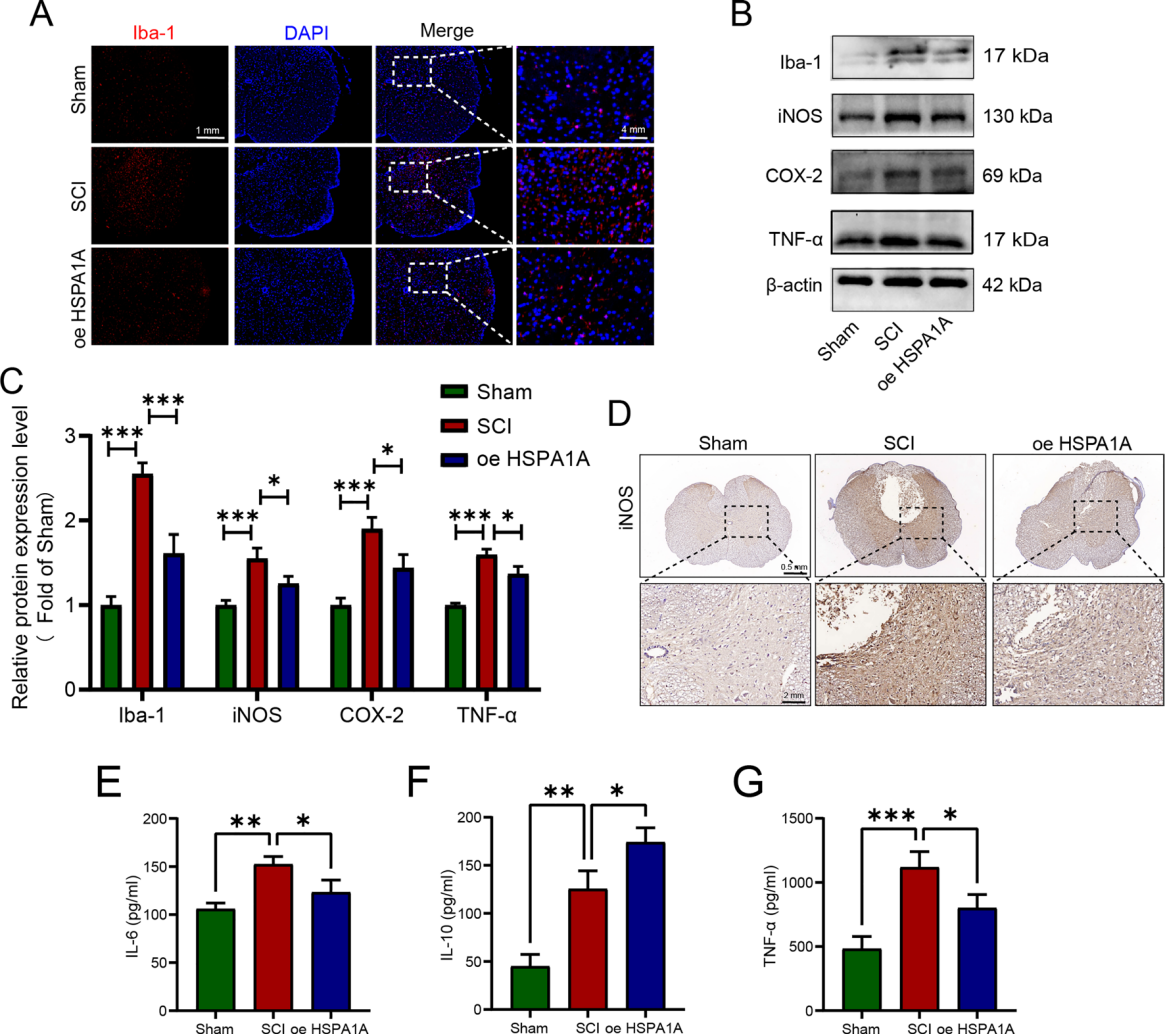
HSPA1A overexpression inhibited inflammation in SCI rats. **A** Representative immunofluorescence images of Iba-1 (red) in the spinal cord on the 7th day after surgery in each group. **B** Western blotting analysis of Iba-1, iNOS, COX-2 and TNF-α expression levels in each group. **C** Quantitative analysis of Iba-1, iNOS, COX-2 and TNF-α levels. **D** Representative immunohistochemistry images of iNOS in the spinal cord at 7 d after surgey in each group. **E** ELISA analysis of IL-6 levels in each group. **F** ELISA analysis of IL-10 levels in each group. **G** ELISA analysis of TNF-α levels in each group. (*P < 0.05, **P < 0.01 and ***P < 0.001)

We performed immunofluorescence to detect the microglial marker Iba-1. As shown in Fig. 3A, there were fewer Iba-1-positive cells in the Sham group, but they showed a significant increase in number in the injury region after SCI, and HSPA1A overexpression reduced the number of Iba-1-positive cells. Western blotting analysis of inflammation-related proteins showed that iNOS, COX-2, and TNF-α expression levels increased significantly after SCI, and HSPA1A overexpression decreased the expression of iNOS, COX-2, and TNF-α (Fig. 3B, C). Immunohistochemical analysis of iNOS was consistent with western blotting analysis (Fig. 3D). The ELISA results showed that HSPA1A overexpression significantly downregulated the levels of TNF-α and IL-6 and increased the levels of IL-10 compared to the SCI group (Fig. 3E–G). That is, HSPA1A inhibited inflammation in SCI rats.

### HSPA1A inhibited LPS- and nigericin-induced microglial pyroptosis

We established pyroptosis and inflammation models with LPS and nigericin using primary microglia extracted from rats, which were identified by immunofluorescence staining for the microglial marker Iba-1 (Fig. S2A), and rat HAPI microglia purchased from YaJi Biological Co., Ltd. The results of both western blotting and ELISA showed that the expression levels of pyroptosis- and inflammation-related proteins were significantly increased after LPS and nigericin induction (Fig. S2, Fig. S3). The above results indicated that LPS and nigericin could successfully model pyroptosis and inflammation in microglia. The results were consistent between primary microglia and HAPI microglia; therefore, we used HAPI microglia instead of primary microglia for subsequent experiments.

**Fig. 4:**
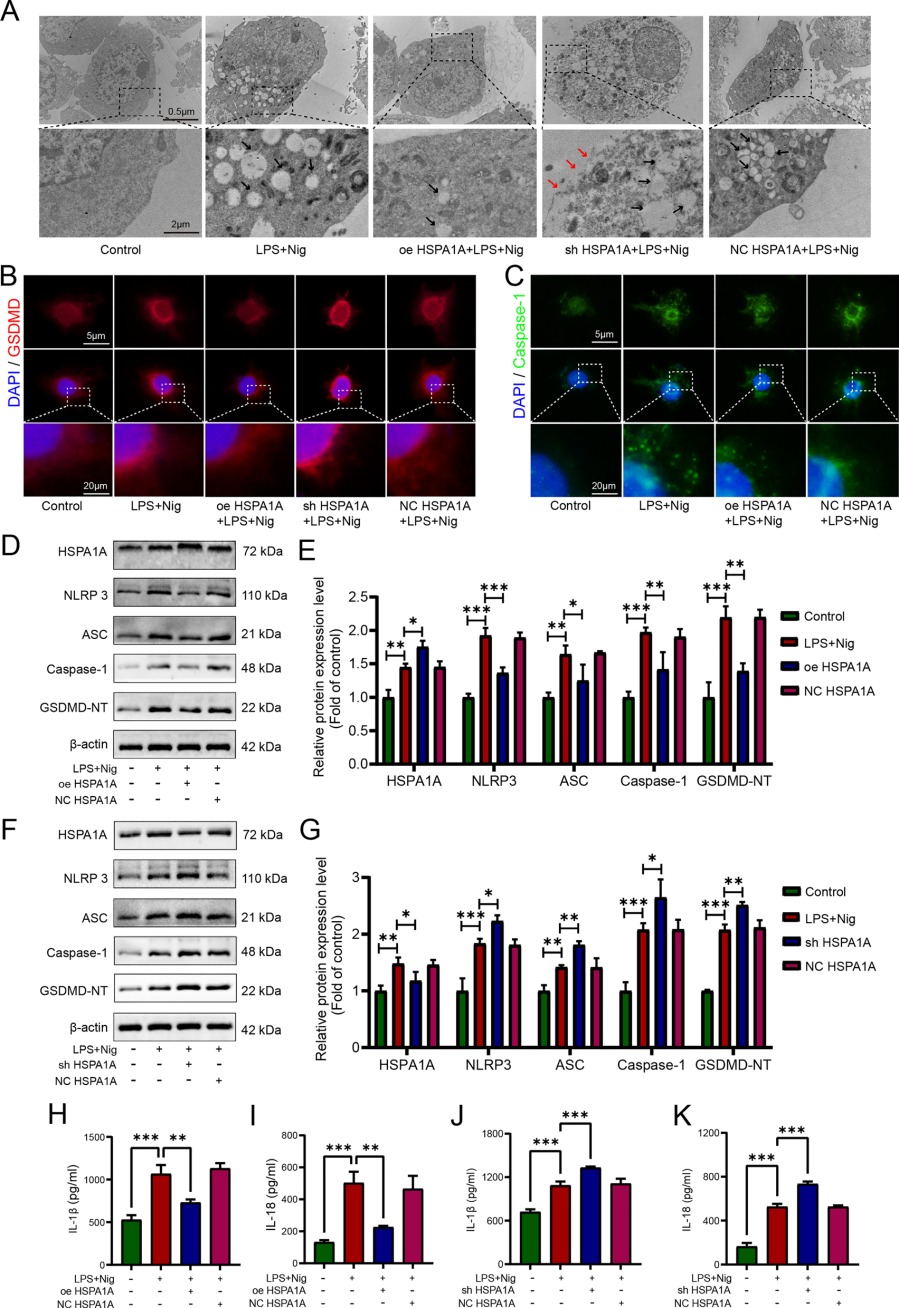
HSPA1A inhibited LPS- and nigericin-induced microglial pyroptosis. **A** Representative transmission electron microscopy images of cells in each group. The black arrow points to the pyroptotic bodies, and the red arrow points to the broken cell membrane. **B** Representative immunofluorescence images of GSDMD (red) in each group. **C** Representative immunofluorescence images of Caspase-1 (green) in each group. **D** Western blotting analysis of NLRP3, ASC, Caspase-1 and GSDMD-NT expression levels in each group. **E** Quantitative analysis of NLRP3, ASC, Caspase-1 and GSDMD-NT levels. **F** Western blotting analysis of NLRP3, ASC, Caspase-1 and GSDMD-NT expression levels in each group. **G** Quantitative analysis of NLRP3, ASC, Caspase-1 and GSDMD-NT levels. **H** ELISA analysis of IL-1β levels in each group. **I** ELISA analysis of IL-18 levels in each group. **J** ELISA analysis of IL-1β levels in each group. **K** ELISA analysis of IL-18 levels in each group. (*P < 0.05, **P < 0.01 and ***P < 0.001)

To explore the effects of HSPA1A on microglial pyroptosis, we established a model of microglial pyroptosis and inflammation induced by LPS and nigericin. Immunofluorescence and western blotting analyses demonstrated successful construction of microglia with HSPA1A overexpression or knockdown by transfection of cells with the corresponding lentiviral vector (Fig. S4A–C). Immunofluorescence analysis showed increased numbers of microglia expressing HSPA1A after LPS and nigericin induction, and that HSPA1A expression was higher in the overexpression group and lower in the knockdown group (Fig. S4D). We then used transmission electron microscopy to observe the changes in cell morphology in each group. As shown in Fig. 4A, pyroptotic bodies appeared in the cells after induction by LPS and nigericin, while they were significantly reduced in the HSPA1A overexpression group compared with the SCI group. Furthermore, LPS- and nigericin-induced pyroptosis was significantly aggravated after knockdown of HSPA1A, with a marked increase in extracellular intracellular pyroptotic bodies and rupture of the cell membrane (Fig. 4A). Immunofluorescence analysis showed that HSPA1A overexpression markedly attenuated LPS- and nigericin-induced expression of the pyroptosis marker proteins Caspase-1 and GSDMD, while HSPA1A knockout enhanced the expression of GSDMD (Fig. 4B, C). Then, we performed western blotting to detect expression of the pyroptosis-related proteins NLRP3, ASC, Caspase-1, and GSDMD-NT in each group. As shown in Fig. 4D and F, the expression levels of NLRP3, ASC, Caspase-1, and GSDMD-NT were significantly increased after LPS and nigericin induction, whereas the levels of these proteins were significantly reduced in the HSPA1A overexpression group compared with the SCI group, and knockdown of HSPA1A resulted in elevated expression of these proteins. Finally, we used ELISA to determine the levels of IL-1β and IL-18 outside the cell. Overexpression of HSPA1A significantly reduced the release of IL-1β and IL-18 induced by LPS and nigericin (Fig. 4H, I), while knockdown of HSPA1A significantly increased the release of IL-1β and IL-18 (Fig. 4J, K). Taken together, these results confirmed that HSPA1A inhibited LPS- and nigericin-induced microglial pyroptosis.

### HSPA1A inhibited LPS- and nigericin-induced microglial inflammation

**Fig. 5:**
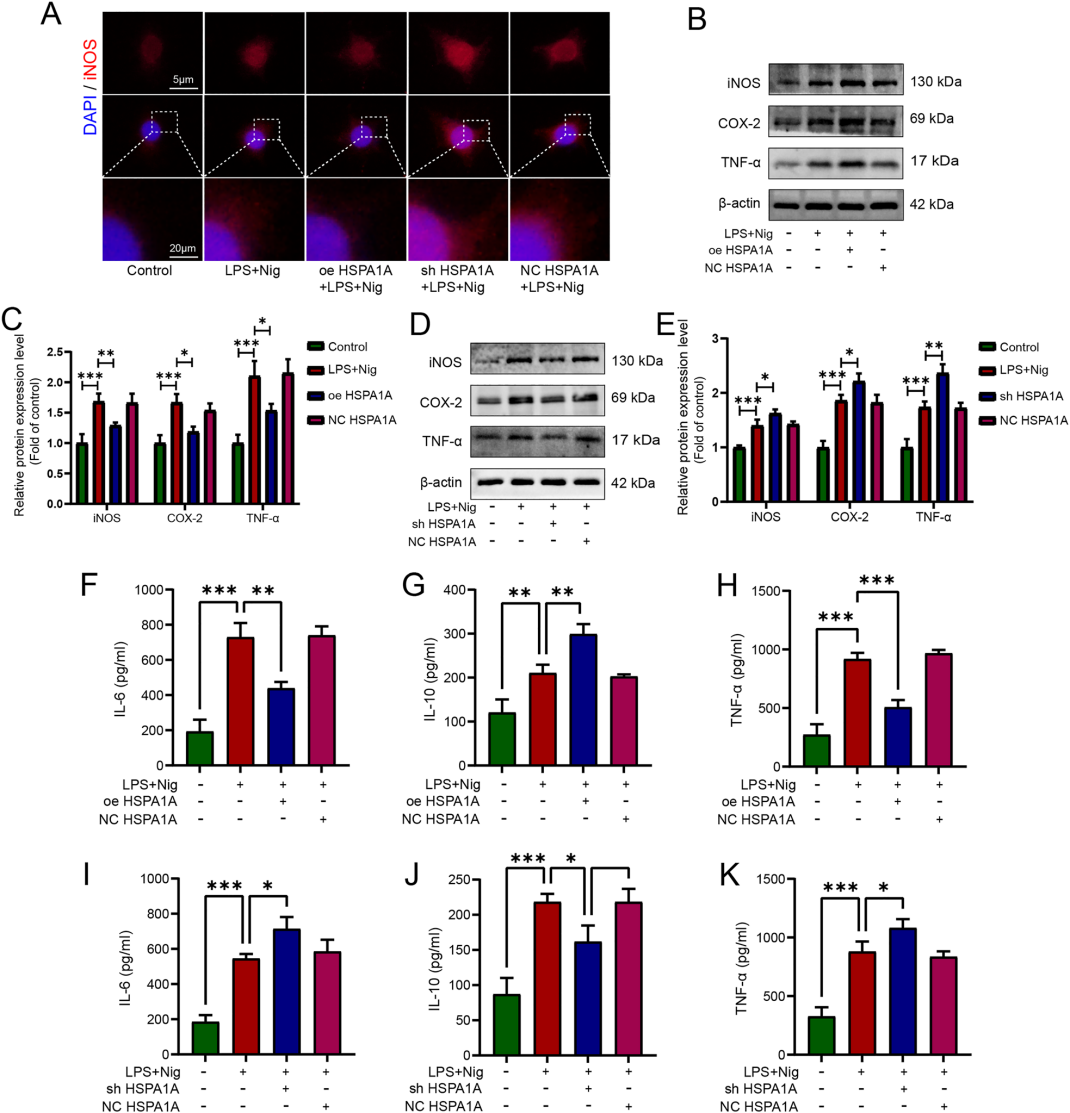
HSPA1A inhibited LPS- and nigericin-induced microglial inflammation. **A** Representative immunofluorescence images of iNOS (red) in each group. **B** Western blotting analysis of iNOS, COX-2 and TNF-α expression levels in each group. **C** Quantitative analysis of iNOS, COX-2 and TNF-α levels. **D** Western blotting analysis of iNOS, COX-2 and TNF-α expression levels in each group. **E** Quantitative analysis of iNOS, COX-2 and TNF-α levels. **F** ELISA analysis of IL-6 levels in each group. **G** ELISA analysis of IL-10 levels in each group. **H** ELISA analysis of TNF-α levels in each group. **I** ELISA analysis of IL-6 levels in each group. **J** ELISA analysis of IL-10 levels in each group. **K** ELISA analysis of TNF-α levels in each group. (*P < 0.05, **P < 0.01 and ***P < 0.001)

We next explored the effects of HSPA1A overexpression or knockdown on LPS- and nigericin-induced inflammation in microglia. Immunofluorescence analysis showed that HSPA1A overexpression markedly attenuated LPS- and nigericin-induced expression of the inflammation marker protein iNOS, while HSPA1A knockout upregulated its expression (Fig. 5A). Next, we performed western blotting to examine expression of the inflammation-related proteins iNOS, COX-2, and TNF-α. As shown in Fig. 5B and D, microglia showed significantly higher levels of iNOS, COX-2, and TNF-α protein expression after induction by LPS and nigericin, whereas these inflammation-related proteins were significantly reduced in the HSPA1A overexpression group and increased in the HSPA1A knockdown group compared with the SCI model group. ELISA results showed that the release of the extracellular inflammatory factors IL-6 and TNF-α and the anti-inflammatory factor IL-10 from microglia was significantly increased by LPS and nigericin induction, and that HSPA1A overexpression markedly decreased LPS- and nigericin-induced IL-6 and TNF-α release and increased IL-10 release (Fig. 5F–H), while knockdown of HSPA1A markedly increased LPS- and nigericin-induced release of IL-6 and TNF-α and decreased IL-10 release (Fig. 5I–K). Taken together, these observations suggested that HSPA1A inhibited LPS- and nigericin-induced microglial inflammation.

### HSPA1A upregulated DUSP1 expression and inhibited MAPK pathway activation

**Fig. 6:**
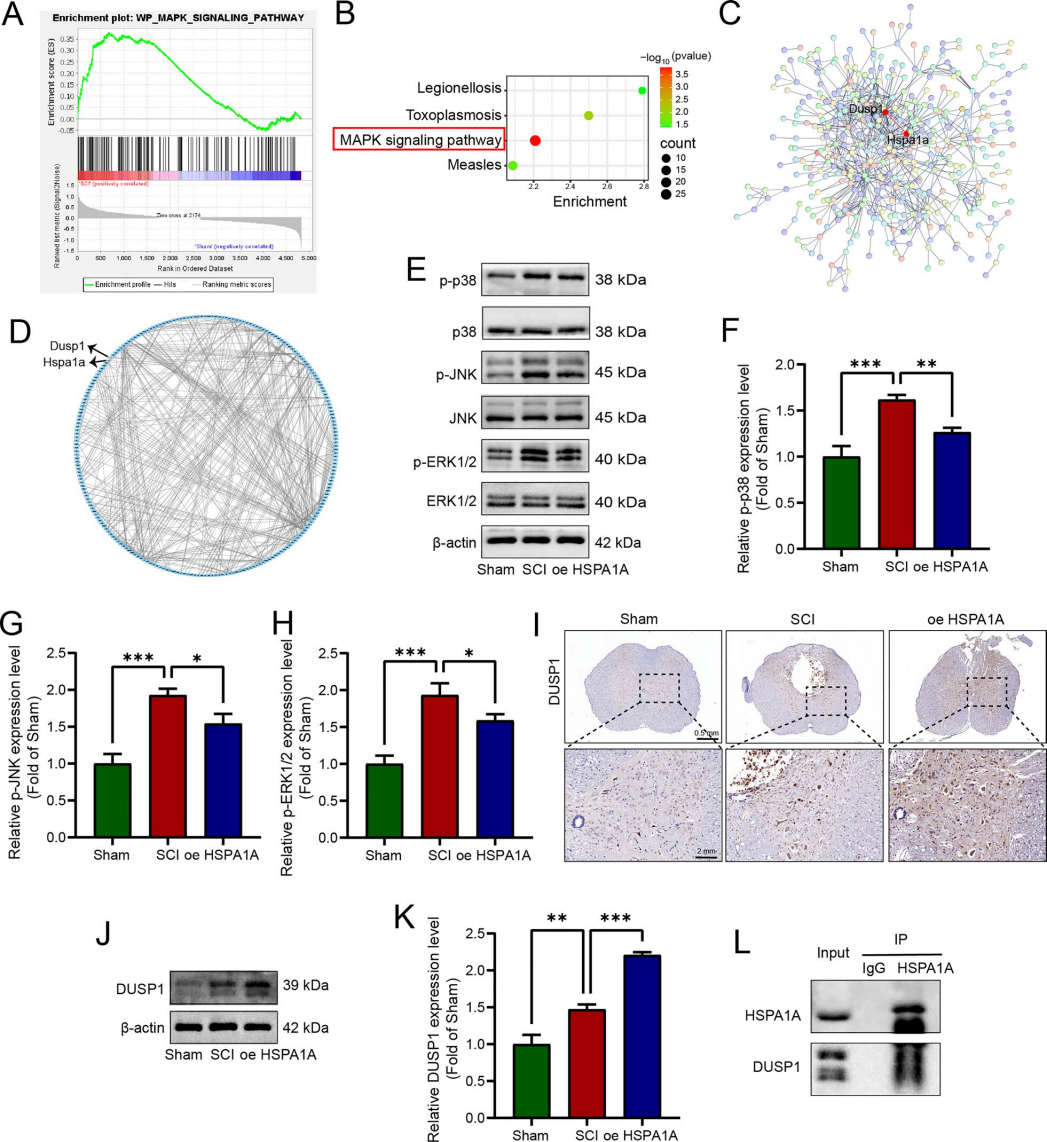
HSPA1A upregulated DUSP1 expression and inhibited MAPK pathway activation. **A** The results of GSEA. **B** Visualization of KEGG results enriched with HSPA1A. **C** PPI networks based on the STRING database. **D** PPI networks analyzed by Cytoscape software. **E** Western blotting analysis of p38, p-p38, JNK, p-JNK, ERK1/2 and p-ERK1/2 levels in each group. **F** Quantitative analysis of p-p38 levels. **G** Quantitative analysis of p-JNK levels. **H** Quantitative analysis of p-ERK1/2 levels. **I** Representative immunohistochemistry images of DUSP1 in the spinal cord on the 7th day after surgery in each group. **J** Western blotting analysis of DUSP1 expression levels in each group. **K** Quantitative analysis of DUSP1 levels. (L) Coimmunoprecipitation analysis of the relationship between HSPA1A and DUSP1. (*P < 0.05, **P < 0.01 and ***P < 0.001)

We performed bioinformatics analyses to further explore the mechanisms underlying HSPA1A-mediated regulation of pyroptosis and inflammation. As described in our previous study, we downloaded the rat SCI dataset GSE464 and performed GSEA, which showed that the MAPK pathway is an important signaling pathway activated after SCI and is involved in the regulation of inflammation (Fig. 6A). Further analyses of DEGs before and after SCI and KEGG analysis of DEGs showed the involvement of HSPA1A in regulation of the MAPK pathway (Fig. 6B). We examined the expression of MAPK pathway-related proteins in rat spinal cord tissues. Western blotting analysis showed that the levels of p-p38, p-JNK, and p-ERK1/2 were significantly elevated after SCI compared with the Sham group, whereas HSPA1A overexpression decreased the levels of p-p38, p-JNK, and p-ERK1/2 (Fig. 6E). The quantitative analysis of western blotting results showed that the phosphorylation levels of p38, JNK and ERK1/2 increased after SCI, but decreased after overexpression of HSPA1A (Fig. 6F, G, H).The relations between the DEGs were analyzed using STRING functional protein association network online data, and the results are shown in Fig. 6C and D. The expression levels of HSPA1A were positively related to those of DUSP1; DUSP1 was also involved in regulation of the MAPK pathway. Next, we examined the expression of DUSP1 in SCI rats by immunohistochemical and western blotting analyses; expression of DUSP1 was elevated after SCI and HSPA1A overexpression further upregulated the expression of DUSP1 (Fig. 6I–K). The results of coimmunoprecipitation experiments indicated the molecular interaction of HSPA1A and DUSP1 (Fig. 6L). In summary, HSPA1A overexpression upregulated DUSP1 expression and inhibited MAPK pathway activation.

### HSPA1A inhibited LPS- and nigericin-induced microglial pyroptosis by upregulating DUSP1 expression and inhibited MAPK pathway activation

**Fig. 7:**
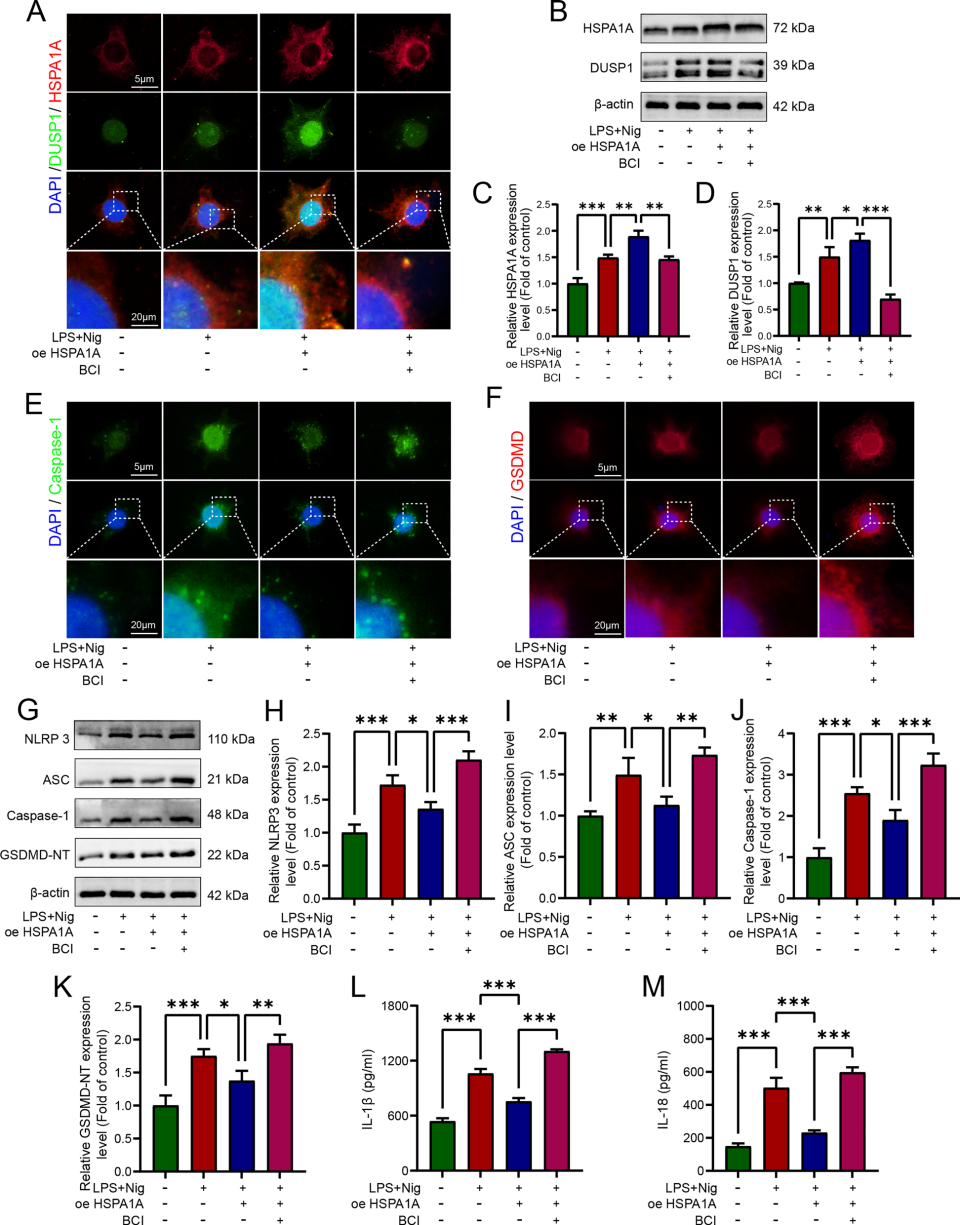
HSPA1A inhibited LPS- and nigericin-induced microglial pyroptosis by upregulating DUSP1 expression and inhibited MAPK pathway activation.** A** Representative immunofluorescence images of HSPA1A (red) and DUSP1 (green) in each group. **B** Western blotting analysis of HSPA1A and DUSP1 expression levels in each group. **C** Quantitative analysis of HSPA1A levels. **D** Quantitative analysis of DUSP1 levels. **E** Representative immunofluorescence images of Caspase-1 (green) in each group. **F** Representative immunofluorescence images of GSDMD (red) in each group. **G** Western blotting analysis of NLRP3, ASC, Caspase-1 and GSDMD-NT expression levels in each group. **H** Quantitative analysis of NLRP3 levels. **I** Quantitative analysis of ASC levels. **J** Quantitative analysis of caspase-1 levels. **K** Quantitative analysis of GSDMD-NT levels. **L** ELISA analysis of IL-1β levels in each group. **M** ELISA analysis of IL-18 levels in each group. (*P < 0.05, **P < 0.01 and ***P < 0.001)

To further clarify the importance of DUSP1 for HSPA1A-mediated regulation of pyroptosis, we inhibited DUSP1 after overexpressing HSPA1A in microglia in vitro. As shown in Fig. 7A and B, HSPA1A overexpression further upregulated the expression of DUSP1. Immunofluorescence analyses showed that inhibition of DUSP1 markedly weakened the inhibitory effect of HSPA1A overexpression on Caspase-1 and GSDMD expression (Fig. 7E and F). Inhibition of DUSP1 significantly attenuated the inhibitory effect of HSPA1A overexpression on Caspase-1 and GSDMD (Fig. 7E, F). Then, we performed western blotting analysis to assess the expression of NLRP3, ASC, Caspase-1, and GSDMD-NT in each group. As shown in Fig. 7G, inhibition of DUSP1 attenuated the inhibition of these pyroptosis-related proteins by overexpression of HSPA1A. Finally, ELISA analysis showed that inhibition of DUSP1 counteracted the inhibitory effect of HSPA1A overexpression on LPS- and nigericin-induced IL-1β and IL-18 release (Fig. 7L, M).

### HSPA1A inhibited LPS- and nigericin-induced microglial inflammation by upregulating DUSP1 expression and inhibited MAPK pathway activation

**Fig. 8:**
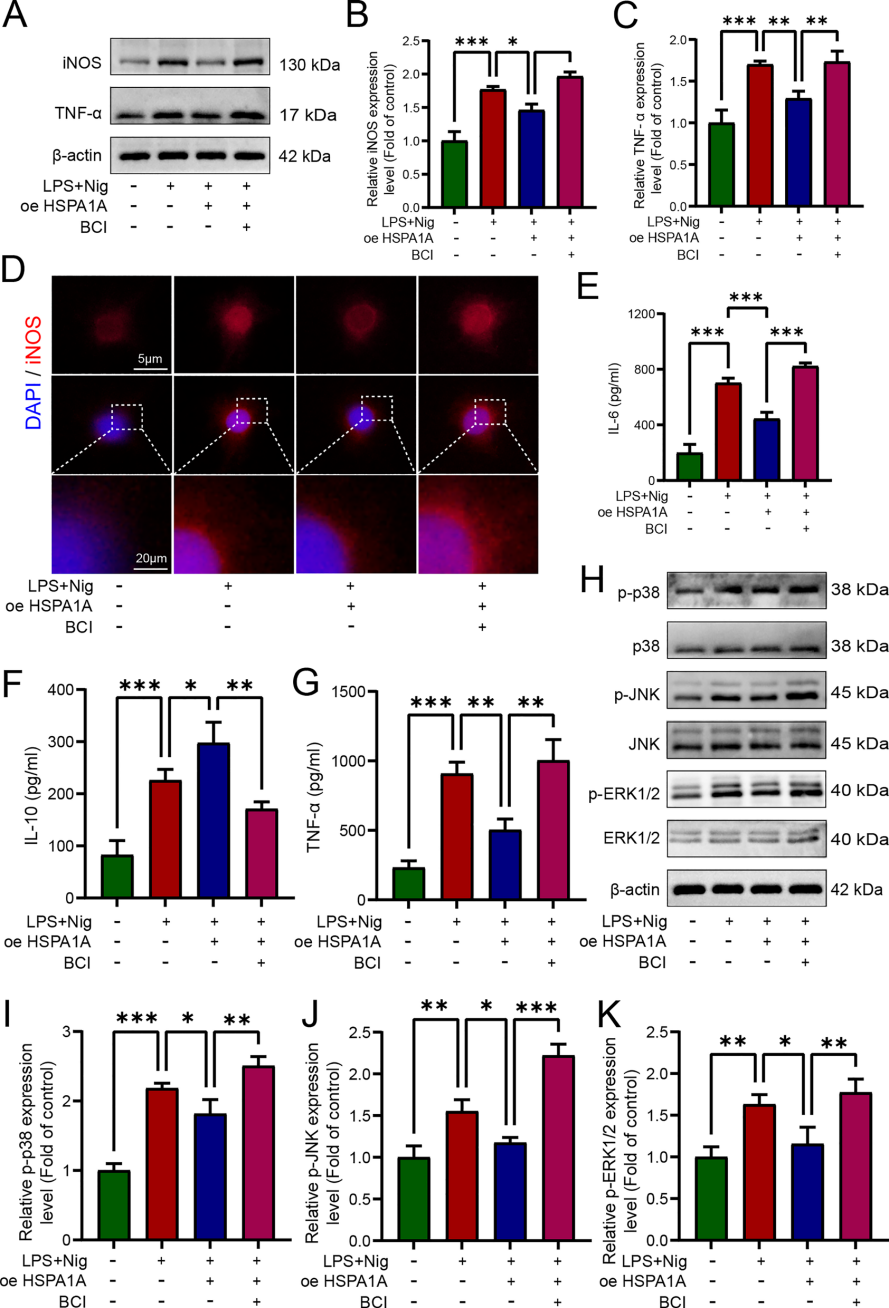
HSPA1A inhibited LPS- and nigericin-induced microglial inflammation by upregulating DUSP1 expression and inhibited MAPK pathway activation. **A** Western blotting analysis of iNOS and TNF-α expression levels in each group. **B** Quantitative analysis of iNOS levels. **C** Quantitative analysis of TNF-α levels. **D** Representative immunofluorescence images of iNOS (red) in each group. **E** ELISA analysis of IL-6 levels in each group. **F** ELISA analysis of IL-10 levels in each group. **G** ELISA analysis of TNF-α levels in each group. **H** Western blotting analysis of p38, p-p38, JNK, p-JNK, ERK1/2 and p-ERK1/2 levels in each group. **I** Quantitative analysis of p-p38 levels. **J** Quantitative analysis of p-JNK levels. **K** Quantitative analysis of p-ERK1/2 levels. (*P < 0.05, **P < 0.01 and ***P < 0.001)

We also explored the role of DUSP1 inhibition in regulation of microglial inflammation by HSPA1A. Western blotting analysis showed that inhibition of DUSP1 significantly attenuated the inhibitory effect of HSPA1A overexpression on iNOS and TNF-α (Fig. 8A). Immunofluorescence analysis of iNOS was consistent with the western blotting (Fig. 8D). ELISA analysis showed that inhibition of DUSP1 significantly increased the release of IL-6 and TNF-α and decreased the release of IL-10 (Fig. 8E–G). Finally, we examined the expression of MAPK pathway-related proteins by western blotting. Overexpression of HSPA1A decreased the levels of p-p38, p-JNK, and p-ERK1/2, whereas levels of these proteins were significantly increased after inhibition of DUSP1 (Fig. 8H). Taken together, these results suggested that HSPA1A inhibitd LPS- and nigericin-induced microglial pyroptosis and inflammation by upregulating DUSP1 expression and inhibiting MAPK pathway activation.

## Discussion

TSCI often leads to permanent neurological dysfunction, which places a tremendous financial burden on patients and medical systems (Cao et al. 2022). However, because of the complexity of the pathophysiology of SCI and the non-reproducible nature of nerve cells, no effective treatments specific for SCI have yet been developed. Therefore, it is important to gain further insights into the underlying mechanisms of SCI, especially the patterns of cell death and inflammation after injury, and to intervene accordingly for treatment. In this study, we identified a significant increase in pyroptosis after SCI. Although HSPA1A has been reported to alleviate inflammation and cell death in several diseases, whether HSPA1A attenuates pyroptosis and inflammation after SCI has not yet been reported. This study showed that HSPA1A improved functional recovery and alleviated pyroptosis in SCI rats. In addition, we further demonstrated that the neuroprotective effect of HSPA1A was exerted through upregulation of DUSP1 to further inhibit the MAPK pathway.

Heat shock proteins (HSPs) are a class of endogenous protective proteins and biomarkers of cellular stress responses that are critical for protein folding, intracellular protein transport, and responses to unfolded and denatured proteins brought about by heat and other stresses (Liu et al. 2022). The 70-kDa HSP family are the most stress-sensitive HSPs, and are widely expressed, highly conserved, and involved in cytoprotection against various types of stress (Havalová et al. 2021). HSPA1A is an important 70-kDa HSP present in the cytoplasm and is highly expressed after SCI (Kabakov and Gabai 2021). HSPA1A is recognized as a general neuroprotective agent that promotes neural repair (Demyanenko et al. 2021). In a cerebral ischemia model, induction of high HSPA1A expression reduced neutrophil infiltration, inhibited the expression of proinflammatory factors, and inhibited apoptosis and glial scar formation (Li et al. 2021). HSPA1A was shown to inhibit the production of proinflammatory factors (TNF-α and IL-6) and inhibit the activity of matrix metalloprotein kinases (MMPs) and iNOS in both in vitro and in vivo models of ischemia (Belenichev et al. 2023). In our study, promoting the expression of HSPA1A increased neuronal survival in the area of the injury, and reduced the number of GFAP-positive cells in SCI rats (Fig. 1). In addition, HSPA1A reduced the activation of microglia at the injury site and inhibited inflammation.

Pyroptosis is significantly increased after SCI. Xu et al. (Xu et al. 2021b) reported significant upregulation of NLRP3 and GSDMD expression in blood samples isolated from patients with SCI. Our previous bioinformatics analysis showed that pyroptosis increased significantly in the acute SCI stage and then decreased gradually in the subacute and chronic stages but remained higher than before injury (He et al. 2023b). In this study, expression of NLRP3, ASC, Caspase-1, GSDMD-NT, IL-1β, and IL-18 increased after SCI in rats, consistent with previous research. After SCI, microglia are rapidly activated, leading to increased expression of proinflammatory factors mediated by multiple signaling cascades, whereas overactivation of microglia leads to overproduction of proinflammatory factors, which in turn exacerbates inflammatory responses and secondary injury (Gaudet and Fonken 2018). In the present study, overexpression of HSPA1A inhibited microglial hyperactivation, pyroptosis, and inflammation after SCI in rats. In vitro*,* HSPA1A overexpression suppressed LPS- and nigericin-induced expression of NLRP3, GSDMD-NT, and the inflammatory factors iNOS and TNF-α, whereas HSPA1A knockdown exacerbated microglial pyroptosis and inflammation. Pyroptotic cells are characterized morphologically as swollen and enlarged cells, with many bubble-like protrusions in the cytoplasm. On electron microscopy, large numbers of vesicles, or pyroptotic bodies, can be seen in the pyroptotic cells. The vesicles then fuse with the cell membrane, forming pores, which cause rupture of the cell membrane, leading to cell death, outflow of cell contents, and release of inflammatory factors (Liu et al. 2021b). The results of the present study showed that HSPA1A overexpression significantly reduced the formation of pyroptotic bodies, whereas knockdown of HSPA1A resulted in a significant increase in pyroptotic bodies and rupture of the cell membrane, which accelerated pyroptosis.

To further investigate the mechanisms by which HSPA1A regulates microglial pyroptosis and inflammation, we performed bioinformatics analysis of rat SCI sequencing data. GSEA showed that the MAPK pathway is an important pathway activated after SCI and is involved in the regulation of inflammation, and KEGG analysis showed that the MAPK pathway was enriched in the HSPA1A gene. It is noteworthy that activation of this pathway is closely related to pyroptosis and inflammation. The proinflammatory effects of the MAPK pathway have been studied extensively, and inhibition of MAPK pathway activation was shown to downregulate the levels of inflammatory mediators after SCI (Xue et al. 2022). In addition, inhibition of the MAPK pathway was reported to shift activated microglial polarization from an M1 proinflammatory phenotype to an M2 anti-inflammatory phenotype, and to reduce the levels of reactive oxygen species (ROS) (Chen et al. 2023b). Recent studies have shown that the MAPK pathway is closely related to pyroptosis. Ri et al. (Ri et al. 2023) demonstrated that Narirutin inhibited NLRP3 inflammasome initiation and NLRP3-ASC interactions in macrophages to inhibit NLRP3 inflammasome activation and further inhibit pyroptosis, which may have been mediated by inhibition of the MAPK pathway. In addition, in a mouse SCI model, elamipretide inhibited pyroptosis by inhibiting phosphorylation of p38 (Zhang et al. 2023). In the present study, the MAPK pathway was activated after SCI, and overexpression of HSPA1A inhibited the levels of p38, JNK, and ERK1/2 phosphorylation.

To further explore how HSPA1A regulates the MAPK pathway, we performed PPI analysis using STRING analysis, which showed that HSPA1A is closely related to DUSP1, a dephosphorylated protein component of the MAPK pathway. Therefore, we examined expression of DUSP1 in rats overexpressing HSPA1A, and both immunohistochemical staining and western blotting indicated that overexpression of HSPA1A upregulated the expression of DUSP1. Co-IP results also indicated an interaction between HSPA1A and DUSP1. DUSP1 is a negative feedback regulator of the MAPK pathway (Kirk et al. 2020), and in vitro and in vivo studies have shown that DUSP1 is an important regulator of innate and adaptive immune responses and inflammation (Scotece et al. 2023). To further explore the role of DUSP1 in HSPA1A-mediated regulation of pyroptosis and inflammation, we inhibited DUSP1 after overexpression of HSPA1A in microglia in vitro. The results showed that inhibition of DUSP1 counteracted the inhibitory effect of HSPA1A overexpression on the expression of pyroptosis-related proteins and the release of inflammatory factors induced by LPS and nigericin; the MAPK pathway was also suppressed. Therefore, we suggest that HSPA1A inhibits activation of the MAPK pathway through DUSP1, which is involved in the regulation of microglial pyroptosis and suppresses neuroinflammation.

Our research points to overexpression of HSPA1A in microglia as a potential therapeutic approach for countering SCI, and its potential mechanism is to inhibit the pyroptosis and inflammation of microglia. However, the role of HSPA1A in other cells, such as neurons, oligodendrocytes and astrocytes, is still unclear, which needs further study. In this study, we used transgenic technology to realize the overexpression of HSPAIA, but it may be safer to use HSPA1A protein or inducer in clinical treatment. In addition, the role of HSPA1A and MAPK pathway is not only to regulate pyroptosis and inflammation, so it is necessary to explore the effects of HSPA1A overexpression and MAPK pathway inhibition on other biological functions, such as cell proliferation and apoptosis. In terms of mechanism, although we have confirmed the interaction between HSPA1A and DUSP1 by co-IP; in the future, we will use gene mice to validate our hypotheses regarding the underlying mechanisms.

## Conclusion

**Fig. 9:**
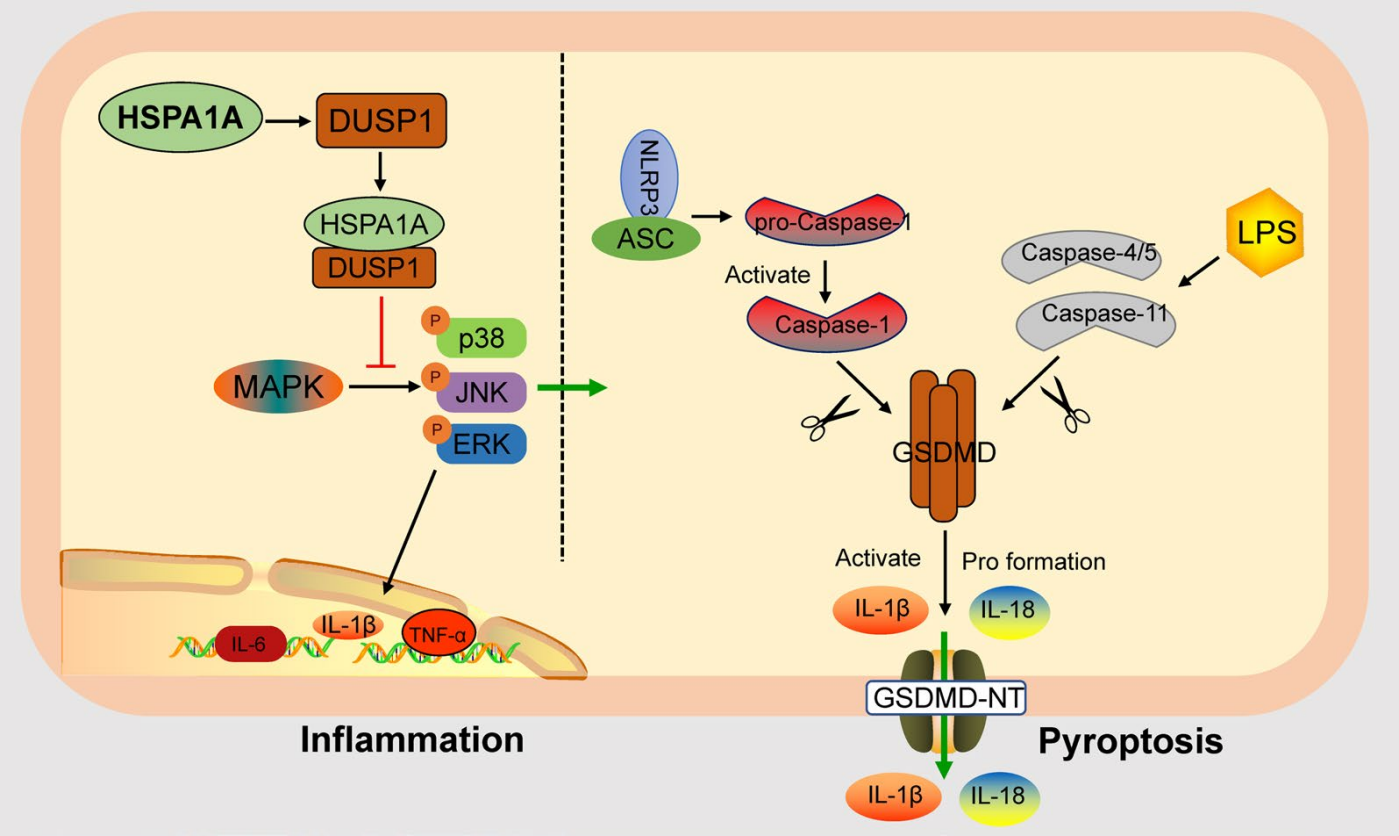
Schematic diagram of HSPA1A inhibiting pyroptosis and neuroinflammation via DUSP1 inhibition of the MAPK signaling pathway

The results of the present study suggest that pyroptosis, the proinflammatory form of cell death, is significantly increased after SCI. HSPA1A inhibited activation of the MAPK pathway by upregulating the expression of DUSP1 (Fig. 9), which suppressed microglial pyroptosis and inflammation. Our results indicate the therapeutic potential of promoting HSPA1A expression in the treatment of TSCI.

## Supplementary Information

Additional file 1.

Additional file 2. Fig. S1 Pyroptosis and inflammation increased in SCI rats. Fig. S2 Establishment of a rat primary microglial model of pyroptosis and inflammation. Fig. S3 Establishment of a rat HAPI microglia model of pyroptosis and inflammation. Fig. S4 Overexpression and knockdown of HSPA1A in microglia.

## Data Availability

No datasets were generated or analysed during the current study.

## Abbreviations

TSCI: Traumatic spinal cord injury
HSPA1A: Heat shock protein family A member 1A
LPS: Lipopolysaccharide
DUSP1: Dual specificity phosphatase 1
MAPK: Mitogen-activated protein kinase
GSDMD: Gasdermin D
Caspase: Cysteinyl aspartate specific proteinases
ASC: Apoptosis-associated speck-like protein containing a CARD
GSDMD -NT: N-terminal fragment of GSDMD
CNS: Central nervous system
ERK: Extracellular signal-regulated kinase
JNK: C-Jun N-terminal kinase
BBB: Basso − Beattie − Bresnahan
HE: Hematoxylin and eosin
BCI: Inhibitor (E)-2-benzylidene-3-(cyclohexylamino)-2,3-dihydro-1H-inden-1-one
ELISA: Enzyme-linked immunosorbent assay
NeuN: Neuronal nuclei antigen
GFAP: Glial fibrillary acidic protein
Iba-1: Ionized calcium binding adaptor molecule 1
iNOS: Inducible nitric oxide synthase
HAPI: Highly aggressively proliferating immortalized
COX-2: Cyclooxygenase 2
PBS: Phosphate-buffered saline
ICH: Immunohistochemical
DBA: 3,3’-Diaminobenzidine
IF: Immunofluorescence
DAPI: 4′,6-Diamidino-2-phenylindole
GSEA: Gene set enrichment analysis
KEGG: Kyoto Encyclopedia of Genes and Genomes
PPI: Protein‒protein interaction
GEO: Gene expression omnibus
FDR: False defection rate
DEGs: Differentially expressed genes
co-IP: Coimmunoprecipitation
DMEM: Dulbecco’s modified Eagle’s medium
FBS: Fetal bovine serum
TEM: Transmission electron microscopy
SEM: Standard error of the mean
ANOVA: Analysis of variance
HSPs: Heat shock proteins
MMPs: Matrix metalloprotein kinases
ROS: Reactive oxygen species
