# Supplementary material for: HSPA1A inhibits pyroptosis and neuroinflammation after spinal cord injury via DUSP1 inhibition of the MAPK signaling pathway
Source: Mol Med. 2025 Feb 9;31:53. doi: 10.1186/s10020-025-01086-9 (PMC11809008; doi:10.1186/s10020-025-01086-9)
Supplement: Supplementary file 1 — Additional file 1. [file 10020_2025_1086_MOESM1_ESM.pdf]

Original western blots

Fig. S1. C

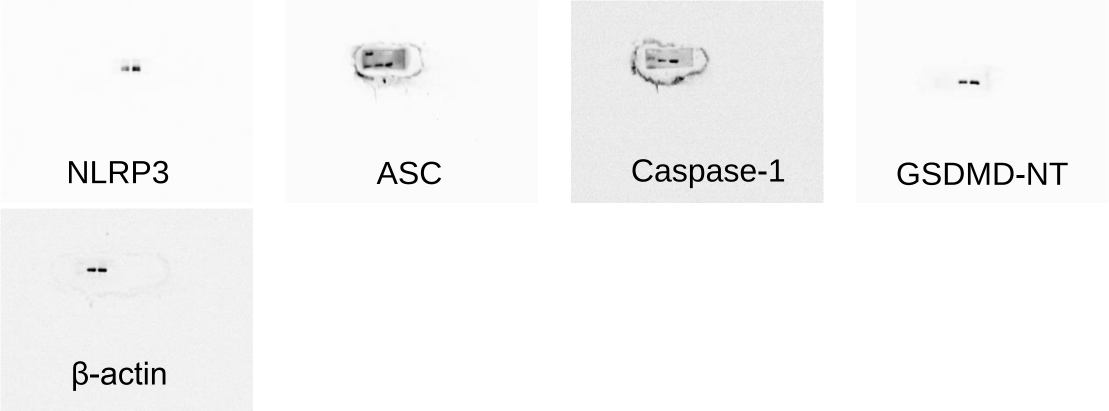

Fig. S1. J

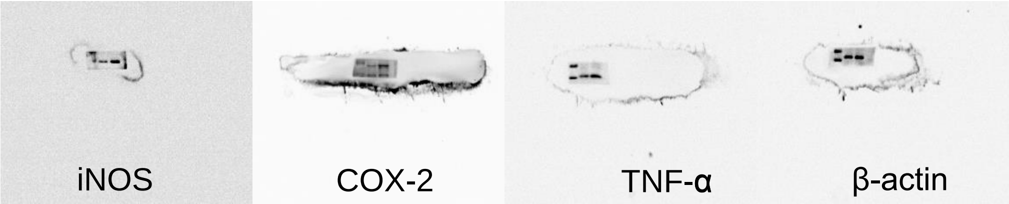

Fig. S2. B

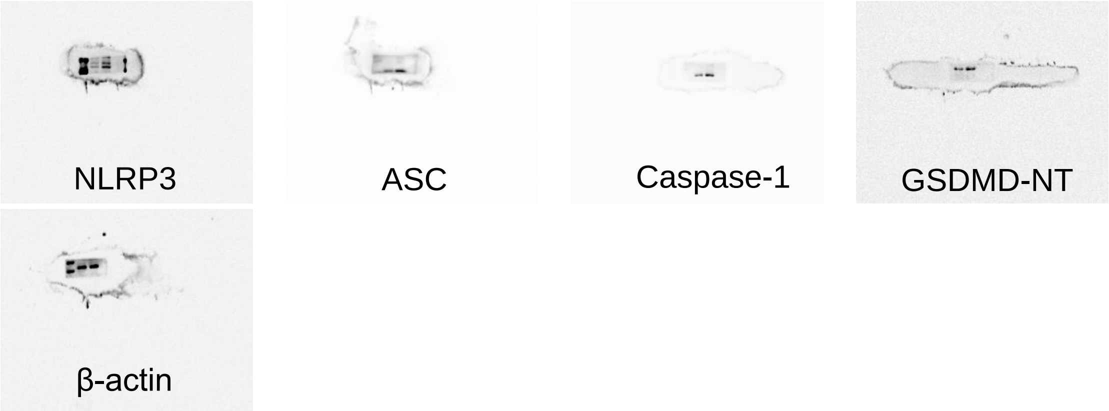

Fig. S2. I

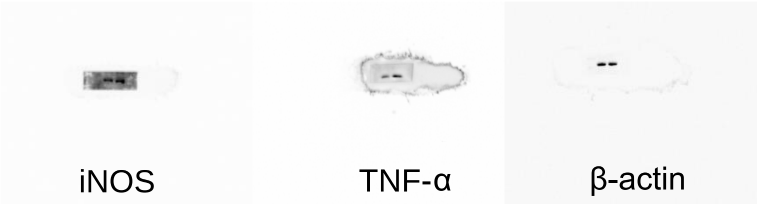

Fig. S3. A

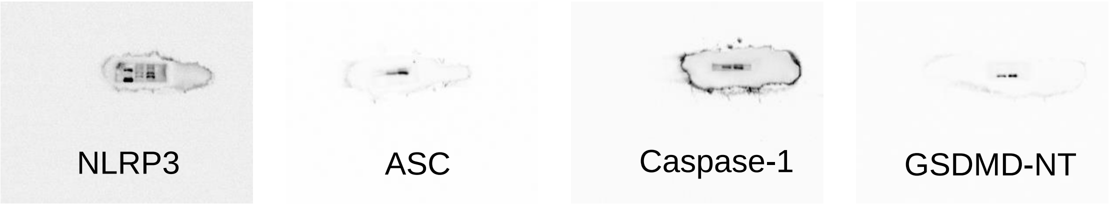

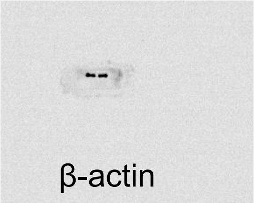

Fig. S3. H

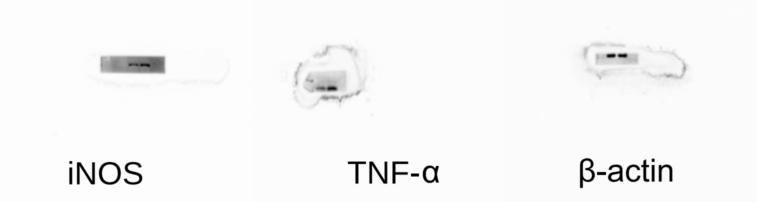

Fig. S4. A

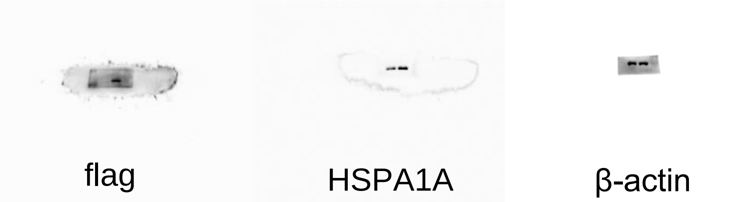

Fig. S4. C

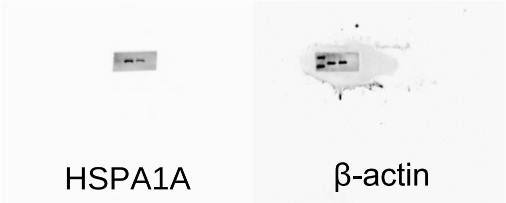

Fig. 1. B

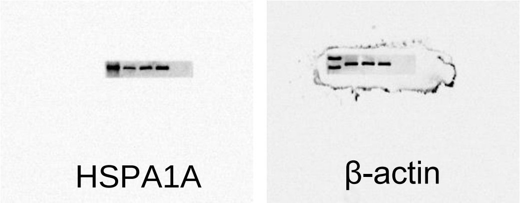

Fig. 1. K

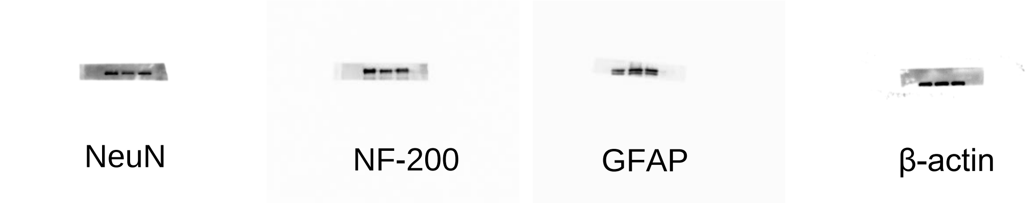

Fig. 2. C

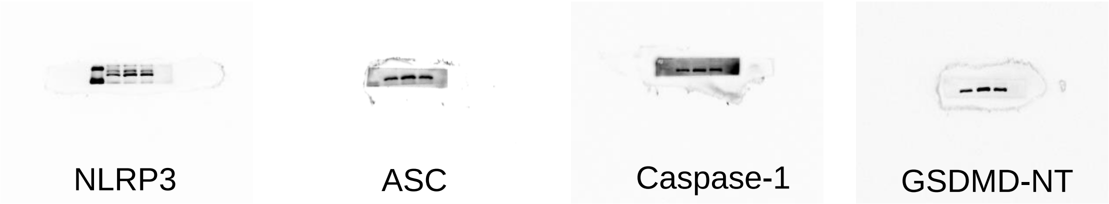

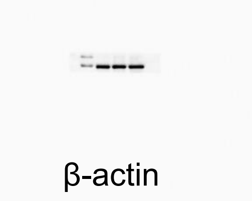

Fig. 3. B

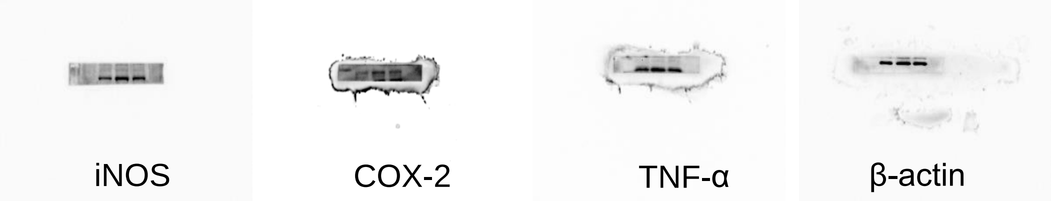

Fig. 4. D

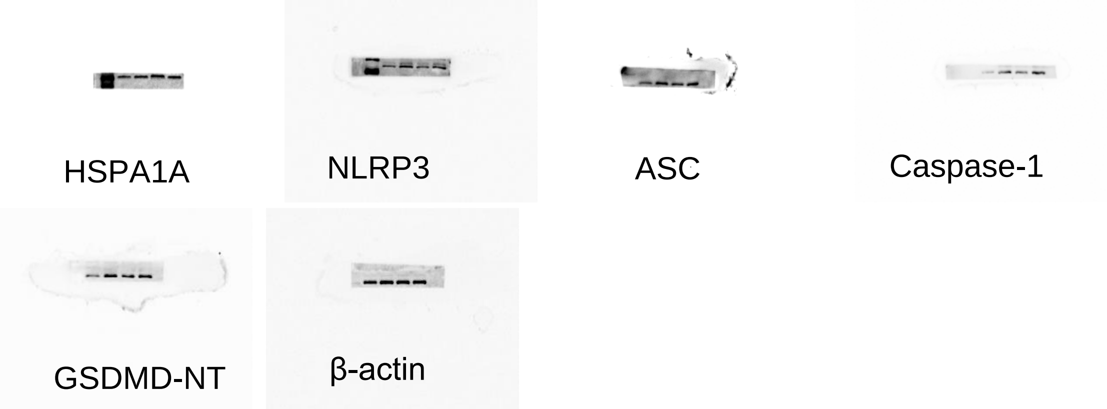

Fig. 4. F

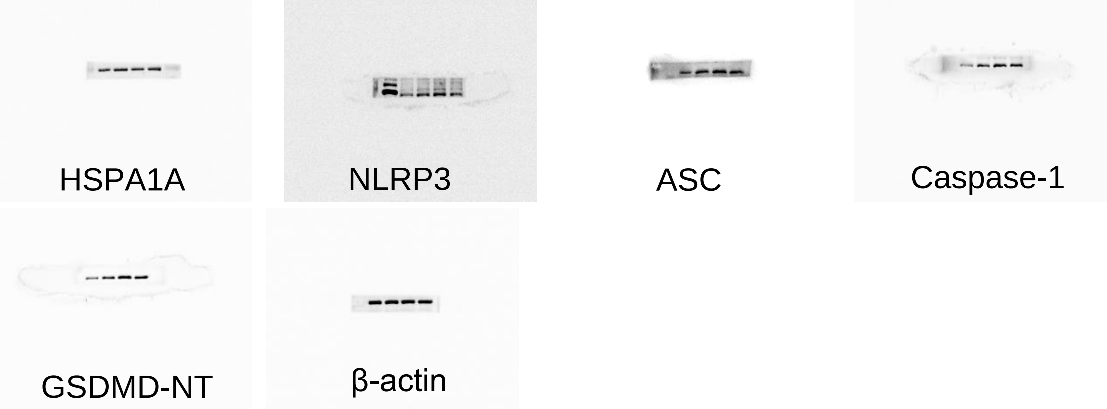

Fig. 5. B

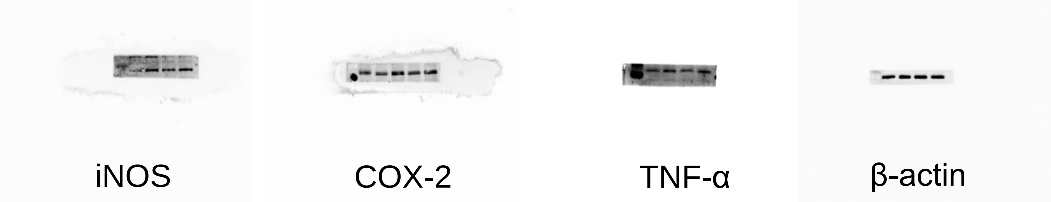

Fig. 5. D

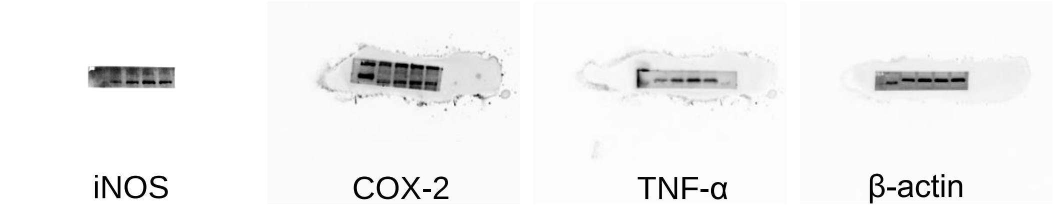

Fig. 6. E

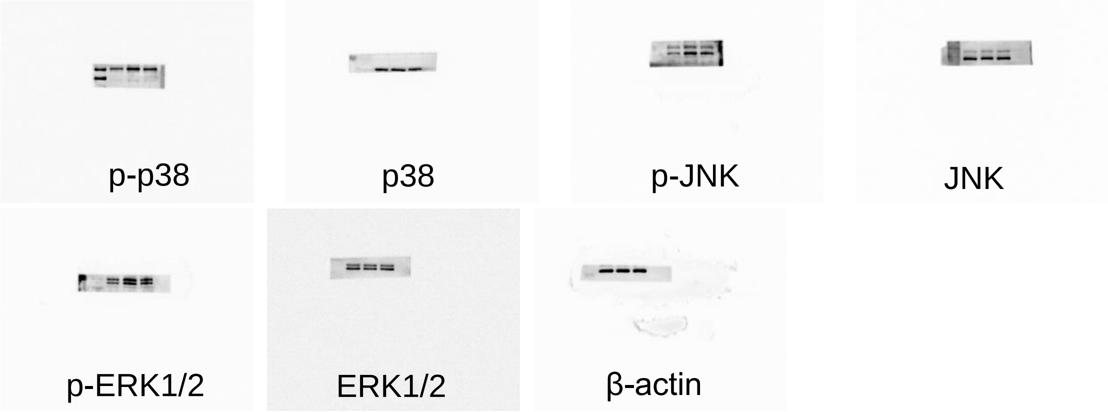

Fig. 6. J

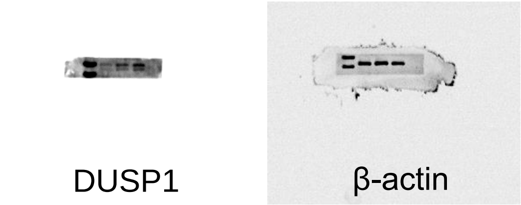

Fig. 6. L

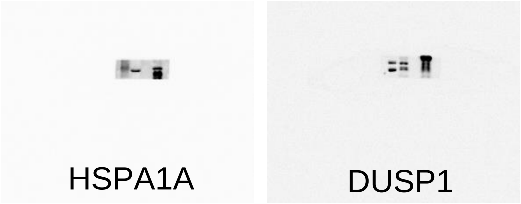

Fig. 7. B

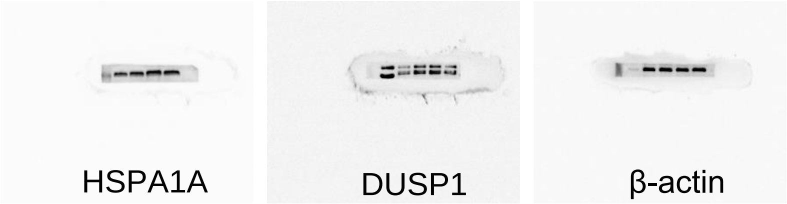

Fig. 7. G

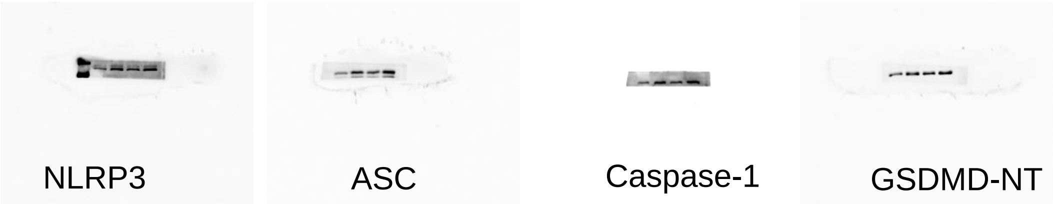

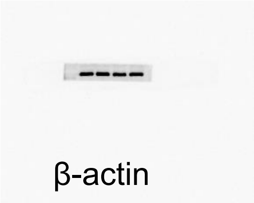
β-actin

Fig. 8. A

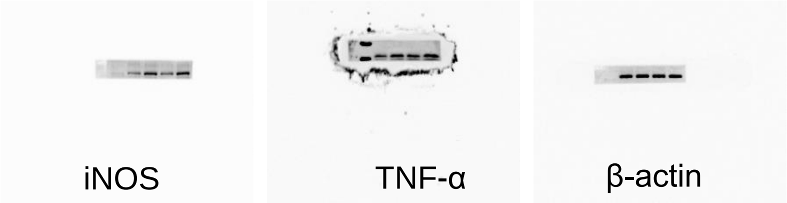
iNOS
TNF-α
β-actin

Fig. 8. H

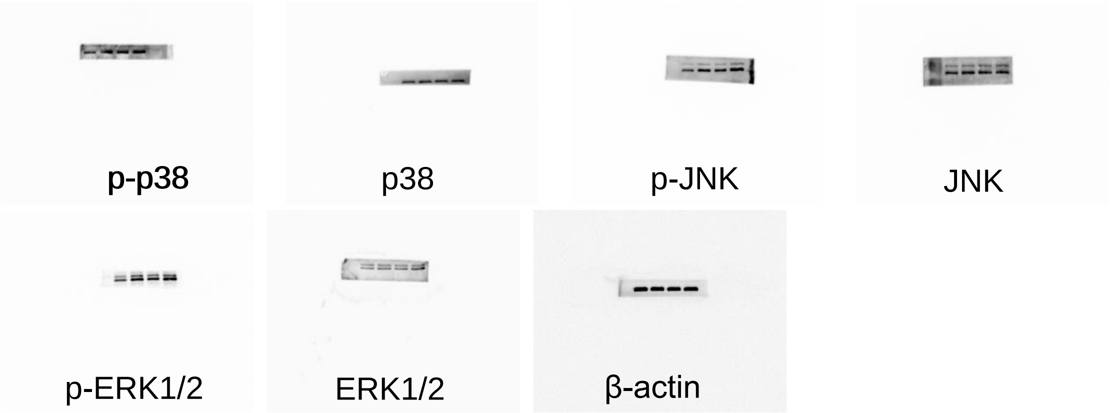
p-p38
p38
p-JNK
JNK
p-ERK1/2
ERK1/2
β-actin
