# Supplementary material for: HSPA1A inhibits pyroptosis and neuroinflammation after spinal cord injury via DUSP1 inhibition of the MAPK signaling pathway
Source: Mol Med. 2025 Feb 9;31:53. doi: 10.1186/s10020-025-01086-9 (PMC11809008; doi:10.1186/s10020-025-01086-9)
Supplement: Supplementary file 2 — Additional file 2. Fig. S1 Pyroptosis and inflammation increased in SCI rats. Fig. S2 Establishment of a rat primary microglial model of pyroptosis and inflammation. Fig. S3 Establishment of a rat HAPI microglia model of pyroptosis and inflammation. Fig. S4 Overexpression and knockdown of HSPA1A in microglia. [file 10020_2025_1086_MOESM2_ESM.docx]

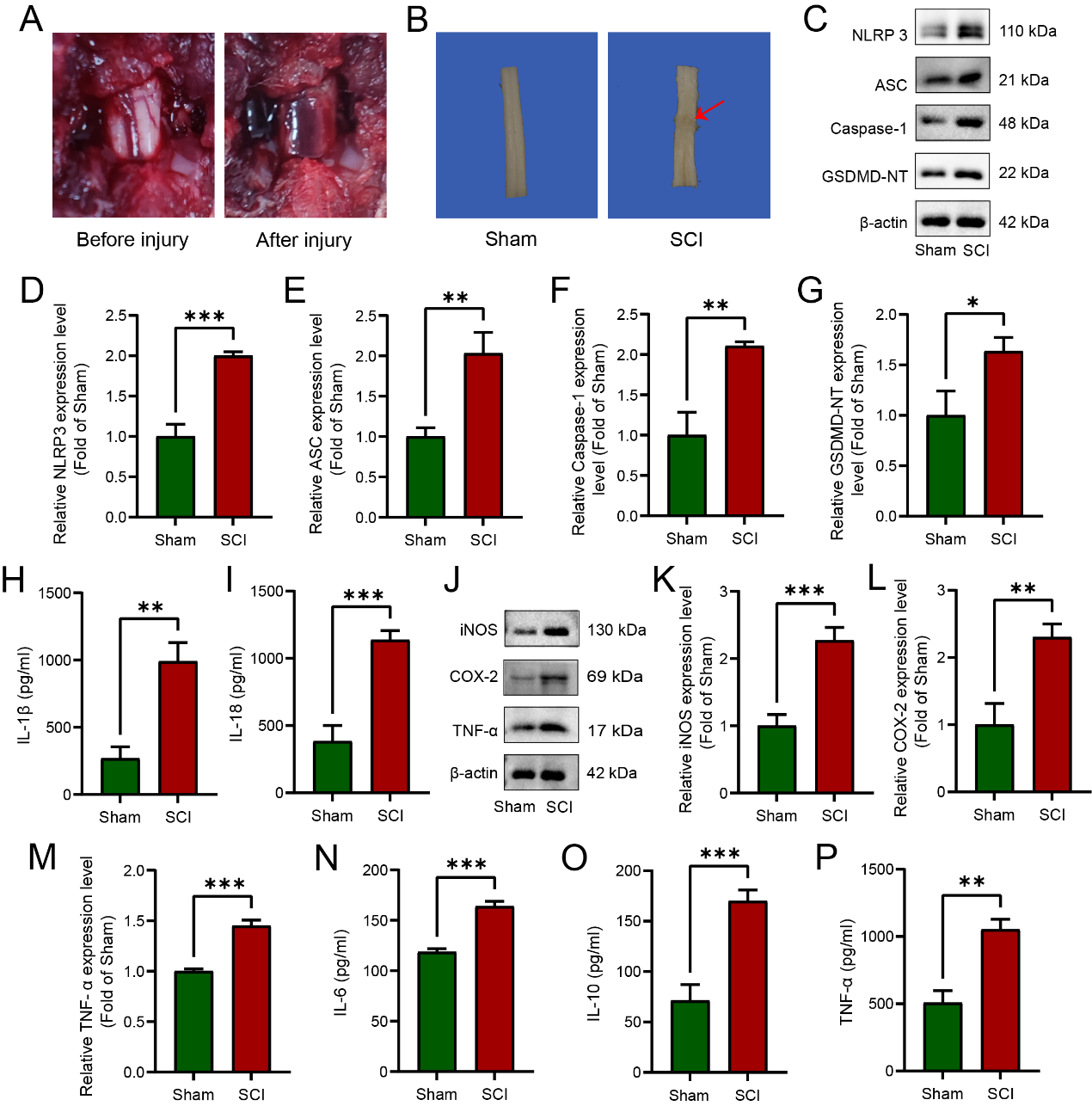


**Fig. S1.** Pyroptosis and inflammation increased in SCI rats. **(A)** Representative spinal cord images of rats before and after SCI. **(B)** Representative spinal cord images of the sham group and SCI group on the 7th day after surgery. The red arrow points to the injury site. **(C)** Western blot analysis of NLRP3, ASC, Caspase-1 and GSDMD-NT expression levels in the sham group and SCI group. **(D)** Quantitative analysis of NLRP3 levels. **(E)** Quantitative analysis of ASC levels. **(F)** Quantitative analysis of caspase-1 levels. **(G)** Quantitative analysis of GSDMD-NT levels. **(H)** ELISA analysis of IL-1β levels in the sham group and SCI group. **(I)** ELISA analysis of IL-18 levels in the sham group and SCI group. **(J)** Western blot analysis of iNOS, COX-2 and TNF-α expression levels in the sham group and SCI group. **(K)** Quantitative analysis of iNOS levels. **(L)** Quantitative analysis of COX-2 levels. **(M)** Quantitative analysis of TNF-α levels. **(N)** ELISA analysis of IL-6 levels in the sham group and SCI group. **(O)** ELISA analysis of IL-10 levels in the sham group and SCI group. **(P)** ELISA analysis of TNF-α levels in the sham group and SCI group. (*P< 0.05, **P < 0.01 and ***P < 0.001).


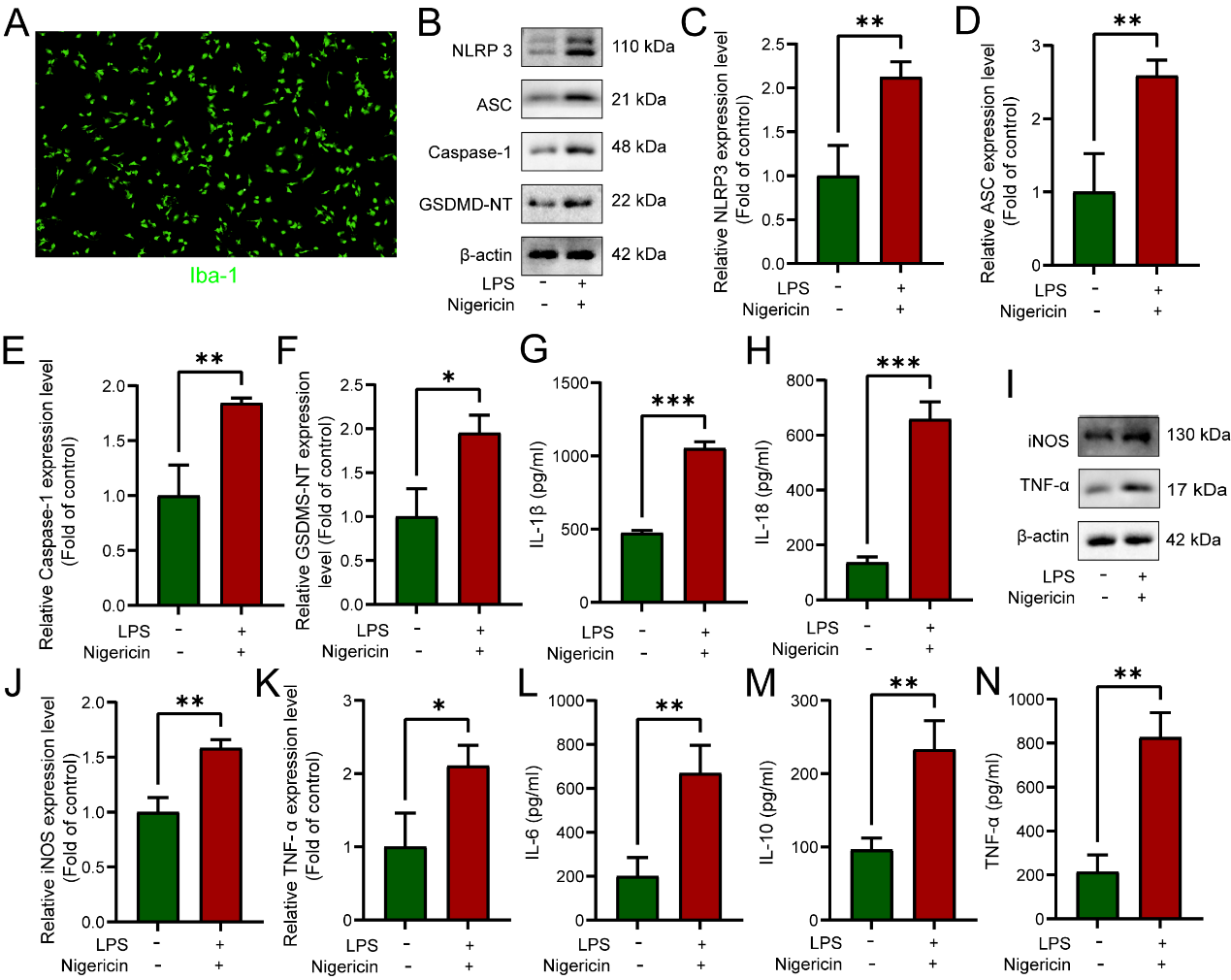


**Fig. S2.** Establishment of a rat primary microglial model of pyroptosis and inflammation. **(A)** Representative immunofluorescence images of Iba-1 (green). **(B)** Western blot analysis of NLRP3, ASC, Caspase-1 and GSDMD-NT expression levels in the control group and model group. **(C)** Quantitative analysis of NLRP3 levels. **(D)** Quantitative analysis of ASC levels. **(E)** Quantitative analysis of caspase-1 levels. **(F)** Quantitative analysis of GSDMD-NT levels. **(G)** ELISA analysis of IL-1β levels in the control group and model group. **(H)** ELISA analysis of IL-18 levels in the control group and model group. **(I)** Western blot analysis of iNOS and TNF-α expression levels in the control group and model group. **(J)** Quantitative analysis of iNOS levels. **(K)** Quantitative analysis of TNF-α levels. **(L)** ELISA analysis of IL-6 levels in the control group and model group. **(M)** ELISA analysis of IL-10 levels in the control group and model group. **(N)** ELISA analysis of TNF-α levels in the control group and model group. (*P< 0.05, **P < 0.01 and ***P < 0.001).


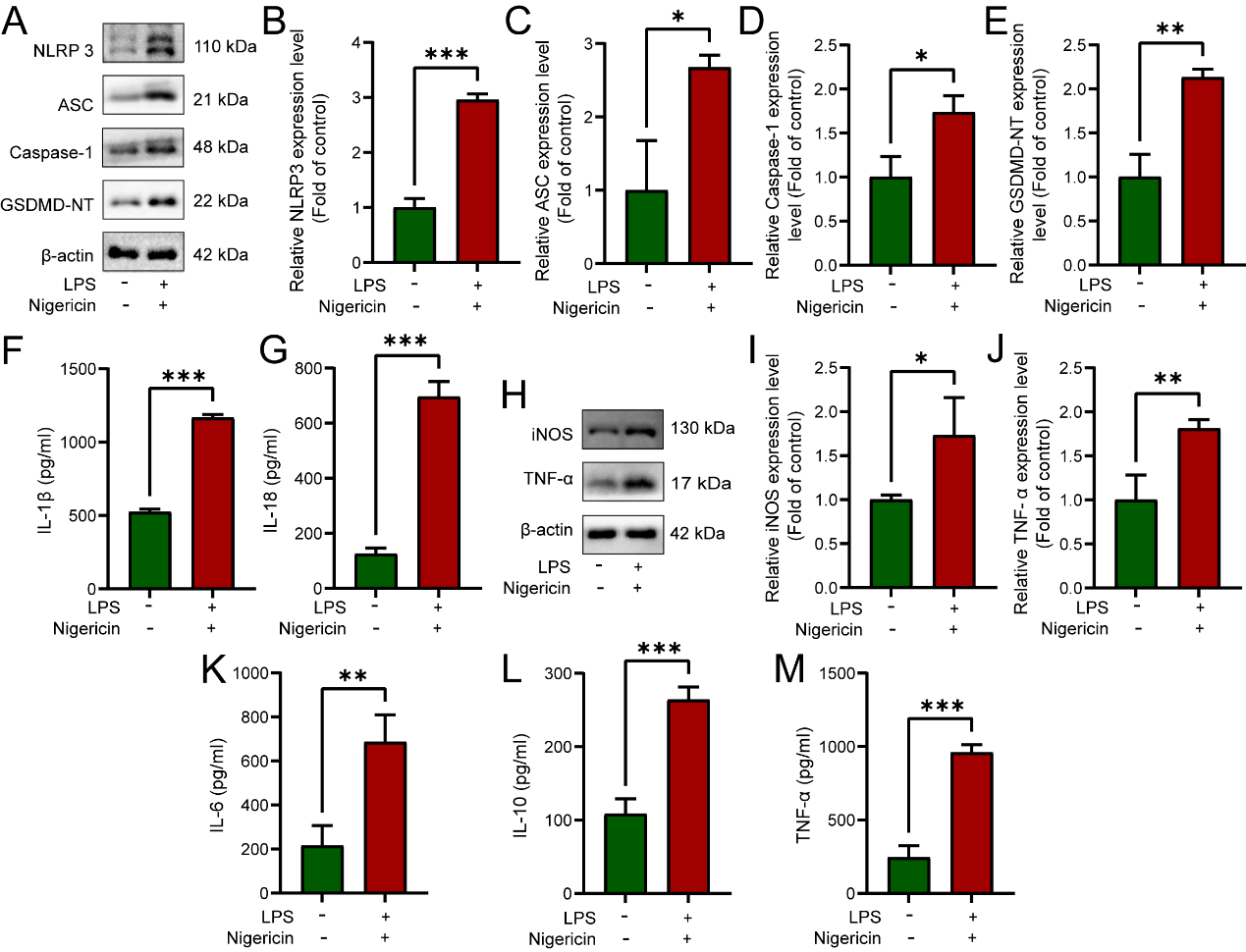


**Fig. S3.** Establishment of a rat HAPI microglia model of pyroptosis and inflammation. **(A)** Western blot analysis of NLRP3, ASC, Caspase-1 and GSDMD-NT expression levels in the control group and model group. **(B)** Quantitative analysis of NLRP3 levels. **(C)** Quantitative analysis of ASC levels. **(D)** Quantitative analysis of caspase-1 levels. **(E)** Quantitative analysis of GSDMD-NT levels. **(F)** ELISA analysis of IL-1β levels in the control group and model group. **(G)** ELISA analysis of IL-18 levels in the control group and model group. **(H)** Western blot analysis of iNOS and TNF-α expression levels in the control group and model group. **(I)** Quantitative analysis of iNOS levels. **(J)** Quantitative analysis of TNF-α levels. **(K)** ELISA analysis of IL-6 levels in the control group and model group. **(L)** ELISA analysis of IL-10 levels in the control group and model group. **(M)** ELISA analysis of TNF-α levels in the control group and model group. (*P< 0.05, **P < 0.01 and ***P < 0.001).


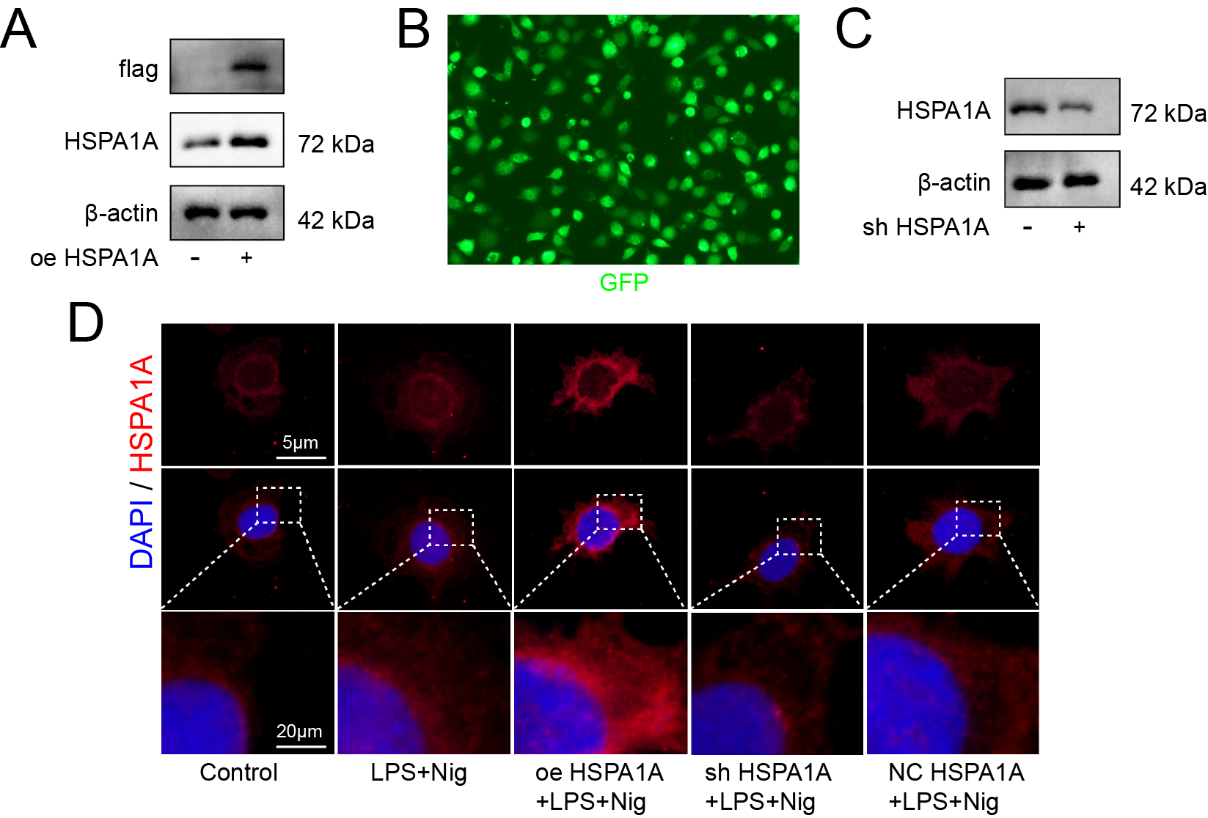


**Fig. S4.** Overexpression and knockdown of HSPA1A in microglia. **(A)** Western blot analysis of HSPA1A protein. **(B)** Representative immunofluorescence images of GFP (green). **(C)** Western blot analysis of HSPA1A protein. **(D)** Representative immunofluorescence images of HSPA1A (red) in each group.
